# Liquid Chromatography-Tandem Mass Spectrometry Analysis of Acetaminophen Covalent Binding to Glutathione *S*-Transferases

**DOI:** 10.3389/fchem.2019.00558

**Published:** 2019-08-13

**Authors:** Timon Geib, Cristina Lento, Derek J. Wilson, Lekha Sleno

**Affiliations:** ^1^Chemistry Department, Université du Québec à Montréal, Montréal, QC, Canada; ^2^Department of Chemistry, The Centre for Research in Mass Spectrometry, York University, Toronto, ON, Canada

**Keywords:** acetaminophen, reactive metabolite, covalent binding, glutathione *S*-transferase, bottom-up proteomics, data-dependent acquisition, high-resolution tandem mass spectrometry, multiple reaction monitoring

## Abstract

Acetaminophen (APAP)-induced hepatotoxicity is the most common cause of acute liver failure in the Western world. APAP is bioactivated to *N*-acetyl *p*-benzoquinone imine (NAPQI), a reactive metabolite, which can subsequently covalently bind to glutathione and protein thiols. In this study, we have used liquid chromatography-tandem mass spectrometry (LC-MS/MS) to characterize NAPQI binding to human glutathione *S*-transferases (GSTs) *in vitro*. GSTs play a crucial role in the detoxification of reactive metabolites and therefore are interesting target proteins to study in the context of APAP covalent binding. Recombinantly-expressed and purified GSTs were used to assess NAPQI binding *in vitro*. APAP biotransformation to NAPQI was achieved using rat liver microsomes or human cytochrome P450 Supersomes in the presence of GSTA1, M1, M2, or P1. Resulting adducts were analyzed using bottom-up proteomics, with or without LC fractionation prior to LC-MS/MS analysis on a quadrupole-time-of-flight instrument with data-dependent acquisition (DDA). Targeted methods using multiple reaction monitoring (MRM) on a triple quadrupole platform were also developed by quantitatively labeling all available cysteine residues with a labeling reagent yielding isomerically-modified peptides following enzymatic digestion. Seven modified cysteine sites were confirmed, including Cys112 in GSTA1, Cys78 in GSTM1, Cys115 and 174 in GSTM2, as well as Cys15, 48, and 170 in GSTP1. Most modified peptides could be detected using both untargeted (DDA) and targeted (MRM) approaches, however the latter yielded better detection sensitivity with higher signal-to-noise and two sites were uniquely found by MRM.

## Introduction

Hepatotoxicity induced by acetaminophen (APAP), a widely used analgesic and antipyretic, presents major health care challenges in Western societies (Fagan and Wannan, 1996; Lee, 2004; Larson et al., 2005; Sivilotti et al., 2005; James et al., 2013). At therapeutic doses, APAP is considered harmless (Mazaleuskaya et al., 2015). However, at higher doses, it can act as a potent hepatotoxin, and cause cell necrosis (McGill et al., 2013) and acute liver failure (Lee, 2004). Liver failure induced by APAP correlates often with overdose or misusage, including chronic overuse (Lee et al., 2008). This often results in difficulties in diagnosis and treatment (Cairney et al., 2016).

**Figure 1:**
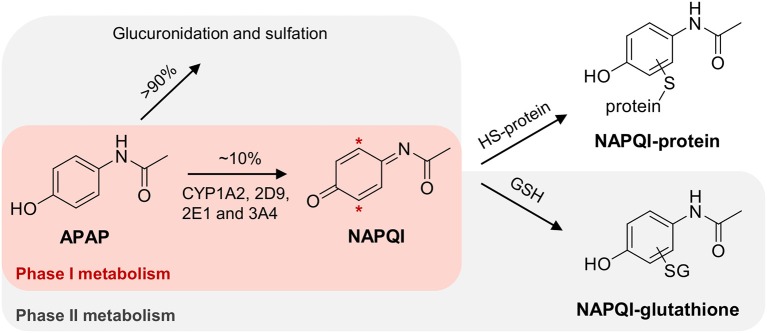
Simplified scheme of APAP metabolism, leading to the formation of NAPQI with subsequent GSH conjugation. Hepatotoxicity of NAPQI is linked to protein binding to cysteine sites.

APAP is mostly detoxified via phase II metabolism; with direct glucuronidation (~55%) and sulfation (~40%) (Mitchell et al., 1973a; Mazaleuskaya et al., 2015). However, up to 10% undergoes bioactivation to *N*-acetyl *p*-benzoquinone imine (NAPQI), a highly reactive electrophile, which is known to covalently bind to nucleophilic centers, including cysteine thiols in liver proteins (see Figure 1) (Jollow et al., 1973; Dahlin et al., 1984; Gibson et al., 1996). At higher doses, the fraction of oxidized APAP can increase up to 15%, due to saturation of the sulfation pathway (Mazaleuskaya et al., 2015). The formation of NAPQI is mediated by the cytochrome P450 (CYP) superfamily (Potter et al., 1973), with CYP1A2, 2D6, 2E1, and 3A4 isoforms as the major APAP oxidizing enzymes (Raucy et al., 1989; Patten et al., 1993; Dong et al., 2000; Manyike et al., 2000; Laine et al., 2009; Preissner et al., 2010; Mazaleuskaya et al., 2015). NAPQI can be detoxified by subsequent phase II metabolism via conjugation with glutathione (GSH), partly mediated by different glutathione *S*-transferase (GST) isozymes (Figure 1) (Mitchell et al., 1973b).

GSTs represent major detoxification enzymes with a protein size of ~25 kDa (Eaton and Bammler, 1999) and multifunctional purposes, including induction of microsomal peroxidation (Strange et al., 2000) and heavy metal resistance (Zhang et al., 2013; Cao et al., 2017), as well as possible roles in diseases, such as pulmonary fibrosis (He et al., 2019). Members of cytosolic GSTs tend to dimerize with subunits of the same class, which results in a larger number of enzyme possibilities than genes (Sheehan et al., 2001). Different genetic variants of GST have shown varying effectiveness in the detoxification of NAPQI. The isozymes GST mu 1, pi 1, and theta 1 (GSTM1, P1, and T1) have been found to be most effective (Coles et al., 1988). However, due to staggered or inadvertent APAP overuse, cellular GSH can be depleted and the GSH conjugation pathway can be saturated, resulting in NAPQI accumulation, leading to increased protein binding (Mitchell et al., 1973b). NAPQI modifications of certain proteins can alter critical cellular functions (Liebler, 2008) and NAPQI-protein adducts have been found to accumulate in hepatocytes (Jollow et al., 1973). Thus, functional and conformational protein changes may ultimately lead to subsequent cell death in liver (Kon et al., 2004). In a previous study, we demonstrated a dose response to protein binding, using absolute quantitation of NAPQI-adducts to Cys34 in rat serum albumin *in vivo* (LeBlanc et al., 2014). A subsequent related study quantifying modified human albumin in patient plasma samples was able to distinguish between moderate and severe APAP-induced acute liver failure (Geib et al., 2018).

Several NAPQI-protein adducts have been identified previously in mouse, rat, and human (Wendel and Cikryt, 1981; Cohen et al., 1997; Qiu et al., 1998; James et al., 2003; Damsten et al., 2007; Copple et al., 2008; Jan et al., 2014). These protein targets consist mostly of cytoplasmic liver proteins, most likely due to the increased solubility and detectability of these proteins. Included in these identified targets is mouse GSTP1 (Qiu et al., 1998). *In vitro* binding studies have also shown NAPQI-GST adducts, using ^14^C-APAP metabolism in mouse liver homogenates (Wendel and Cikryt, 1981). In a previous study in rat liver microsomes (RLM), we reported that rat microsomal GST 1 (MGST1) was a target of NAPQI (Golizeh et al., 2015). A subsequent paper confirmed rat MGST1 as well as GSTM1 as targets (Leeming et al., 2017). Binding to human MGST1 was observed by Shin et al. (2007) studying human liver microsomes incubations of APAP. Boerma et al. (2011) found recombinant human GSTP1 modified after NAPQI formation by a CYPBM3 mutant *in vitro*. Certain of these previous reports were able to pinpoint the specific cysteine residues which were modified in the GSTs, namely Cys50 in rat and human MGST1 (Shin et al., 2007; Golizeh et al., 2015), and Cys48 in human GSTP1 (Boerma et al., 2011). All of these cysteines are significant for enzyme function and alkylation would therefore result in inhibition (Lemercier et al., 2004; Shin et al., 2007; Jenkins et al., 2008). Since GSTs play a significant role in detoxification, further research into the susceptibility of different GST isozymes to being modified by NAPQI remains of high interest.

The analysis of proteins and their modifications benefits from continual advancements in liquid chromatography-tandem mass spectrometry (LC-MS/MS), a broadly used tool for proteomic investigations (Aebersold and Mann, 2003). Constant improvements in sensitivity and selectivity have made LC-MS/MS an invaluable method to characterize proteins and study their post-translational modifications (Aebersold and Mann, 2016). The development of high-resolution mass spectrometry (HRMS) (Mehmood et al., 2015), data-dependent acquisition (DDA) (Mann et al., 2001), targeted multiple reaction monitoring (MRM) (Picotti and Aebersold, 2012; Gillette and Carr, 2013), and multidimensional LC (Di Palma et al., 2012) have all contributed significantly to MS-based proteomics for highly sensitive detection of proteins and their modifications. As a result of its unique ability to distinguish changes in exact sites in the protein sequence (Larsen et al., 2006), MS/MS has become especially vital in post-translational modification studies.

**Figure 2:**
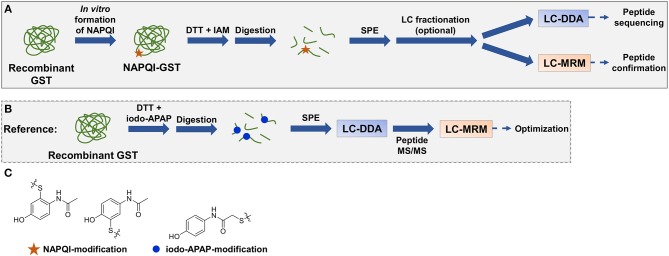
Representative workflow of *in vitro* NAPQI binding to recombinant GSTs followed by reductive alkylation and digestion (trypsin or pepsin), SPE with optional LC fractionation, and LC-MS/MS analysis **(A)**. NAPQI generation was achieved by activation of APAP with either RLM or CYP3A4 Supersomes. MRM methods were based on isomeric iodo-APAP-GST standard, digested and first analyzed by DDA to investigate ionization and fragmentation properties of iodo-APAP-peptides **(B)**. Then, MRM transitions were built and optimized for each peptide individually. Cysteine alkylation reagent iodo-APAP yields positional isomer label to NAPQI-modified cysteine **(C)**.

In this work, several analytical approaches (Figure 2) were investigated combining different techniques for sample preparation and LC-MS/MS analysis to identify APAP-related covalent binding to purified human GSTs (overview in Table 1, note: Met1 is considered here as the first amino acid in protein sequences throughout the whole manuscript). Strength of our study was not needing to use radioactivity or a fluorescence tag to selectively detect modified proteins or peptides (a brief overview of protein detection methods can be found in Table S1). *In vitro* NAPQI-protein binding to four GSTs was investigated (GST alpha 1 (A1), M1, M2, and P1). APAP oxidation was performed *in vitro* using RLM or CYP3A4 Supersomes. Then, NAPQI-modified proteins were digested via two proteases (trypsin and pepsin) in parallel and analyzed by two-dimensional LC-MS/MS, using high-pH reversed-phase (RP) offline fractionation. Results of fractionated and non-fractionated samples were compared. MS/MS was employed with DDA on a high-resolution quadrupole-time-of-flight platform. High-sensitivity MRM measurements were then used for targeted analysis of modified peptides, using reference standards of all possible cysteine modified peptides.

**Table 1:** Overview of studied human GST enzymes^a^.

**GST**	**Accession number**	**Molecular weight [Da]**	**pI**	**Active site (function)**	**Cysteine site**	**References**
A1	P08263	25,631	8.22	Tyr9 and Arg45 (GSH binding)	Cys112	Balogh et al. (2009)
M1	P09488	25,712	5.99	Lys50 (GSH binding) and Tyr116 (substrate binding)	Cys78, 87, 115, and 174	Johnson et al. (1993); Patskovsky et al. (2006)
M2	P28161	25,745	5.79	Lys50 (GSH binding), Tyr116 (substrate binding), and Thr210 (substrate specificity)	Cys87, 115, and 174	Johnson et al. (1993)
P1	P09211	23,356	5.29	Tyr8, Arg14, Trp39, and Lys45 (GSH binding)	Cys15, 48, 102, and 170	Ji et al. (1994); Oakley et al. (1997a,b); Prade et al. (1997); Ang et al. (2009); Federici et al. (2009)

a *Met1 is considered here as first entry in the amino acid sequence*.

## Materials and Methods

### Chemicals and Materials

Aroclor 1254-induced male Sprague-Dawley rat liver microsomes (RLM) (part no.: M10001, lot no.: QEB and OTS) were purchased from BioreclamationIVT (Baltimore, MD). Human CYP3A4 Supersomes (also containing oxidoreductase and cytochrome *b*_5_, part no.: 456202, lot no.: 7304001) were from Corning (Corning, NY). Cysteine alkylating agent iodo-APAP (*N*-(4-hydroxyphenyl)-2-iodoacetamide) was synthesized in-house as previously described (LeBlanc et al., 2014). *Escherichia coli* cells were from laboratory stocks. Magnesium chloride and potassium phosphate (dibasic) were from Anachemia (Montréal, QC). Trypsin (TPCK-treated, from bovine pancreas), pepsin (from porcine gastric mucosa), dithiothreitol (DTT), acetaminophen (APAP), HPLC-grade acetonitrile (ACN) and methanol (MeOH), iodoacetamide (IAM), glucose-6-phosphate dehydrogenase (type XV, from baker's yeast), and other chemicals were purchased from Sigma-Aldrich (St. Louis, MO). Ultrapure water was from a Millipore Synergy UV system (Billerica, MA).

### GST Expression and Purification Protocols

Recombinant human GSTA1, M1, M2, and P1 proteins were expressed in *E. coli* and purified using a modification of published protocols (Mukanganyama et al., 2002; Groom et al., 2014). Overnight induction was proceeded when the culture reached OD_600_ = 0.6. Cells were lysed as previously described (Mukanganyama et al., 2002). Protein concentration was determined with the Bradford assay, using bovine serum albumin as reference standard (Habig et al., 1974). GST affinity purification was performed on a GSH-agarose column (Groom et al., 2014). Purified GSTs were dialyzed to remove GSH by 3 L changes against 500 μL into ammonium acetate for 48 h. Proteins were stored at −80°C.

### Preparation of Alkylated GSTs Using iodo-APAP or IAM

Reductive alkylation of human GSTA1, M1, M2, or P1 (1.8–3.6 nmol) was performed in 100 mM ammonium bicarbonate (ABC, pH 8.5) using DTT (250 nmol; 20 min, 37°C), and either iodo-APAP (750 nmol in ACN; 45 min,) or IAM (750 nmol; 30 min) at 37°C (in dark). Alkylated proteins were incubated with either 10 μg trypsin (4 h at 37°C) in ABC buffer, or 10 μg pepsin (1 h at 37°C) with added 1% formic acid in 10% MeOH to obtain pH 2 during digestion. Trypsin digests were diluted with water; pepsin digests with ABC (100 mM), prior to solid-phase extraction (SPE) on 1 cc (30 mg) OASIS HLB cartridges (Waters, Milford, MA) eluted with 100% MeOH (1 mL). Eluates were dried and then resolubilized in 100 μL of 10% ACN prior to LC-MS/MS analysis.

### NAPQI-GST Formation by RLM Incubation

RLM (1 mg/mL protein) and APAP (100 μM) were incubated (37°C and 500 rpm) in the presence of NADP^+^ (500 μM), and a NADPH-regenerating system: glucose 6-phosphate (10 mM), glucose-6-phosphate dehydrogenase (2 U/mL) and magnesium chloride (5 mM). Human GST (A1, M1, M2, or P1) in 100 mM phosphate buffer (pH 7.4, 1.8–3.6 nmol protein amount) was added to react with produced NAPQI for 3 h. The final incubation volume was 200 μL. Then, proteins were diluted in ABC (100 mM); 200 and 150 μL for tryptic and peptic digestion, respectively.

### NAPQI-GST Formation by CYP3A4

CYP3A4 Supersomes (1 mg/mL protein, 125 pmol/mL CYP3A4 final concentration) and APAP (100 μM) were incubated (37°C and 500 rpm) in the presence of NADPH (1 mM in phosphate buffer). Then, GST (same as above) was added to react with produced NAPQI and diluted after 3 h (as above).

### Digestion of NAPQI-GST

Reductive alkylation was performed using 10 μL DTT (25 mM; 20 min, 37°C) and 10 μL IAM (63 mM, 30 min, 37°C, in dark). All samples were then digested with either 10 μg trypsin (4 h at 37°C), or with 400 μL solution of 1% formic acid in 10% MeOH with 10 μg pepsin (1 h at 37°C). Trypsin digestion was diluted with 500 μL water, pepsin digestion with 200 μL ABC (100 mM). Digests were then cleaned-up by SPE and reconstituted as above.

### High-pH RP Peptide Fractionation

Dried extracts were reconstituted in 10% ACN (120 μL) for injection (100 μL) onto a ZORBAX Extend-C18 column (250 × 4.6 mm; Agilent Technologies, Palo Alto, CA) with 5 μm (100 Å) particles on an Agilent 1200 series HPLC equipped with a temperature-controlled autosampler (at 8°C), binary pump, degasser, diode array detector, and cooled fraction collector (at 8°C). High-pH RP fractionation was performed at a flow rate of 0.6 mL/min with a gradient starting at 5% B for 2 min, increased to 50% B in 12 min, to 70% B within 0.5 min, and held for 6.5 min. Mobile phase A was 10 mM ammonium acetate (pH 10, adjusted with ammonium hydroxide) in water and mobile phase B was prepared with 10% A / 90% ACN. UV absorbance was monitored at 220 and 280 nm. Then, 16 (1 min) fractions were collected from 3 to 19 min. Fractions were concatenated (fraction 1+9, 2+10, …) into eight final fractions, dried and reconstituted in 100 μL 10% ACN.

### LC-MS/MS Analysis With DDA

Samples were injected (30 μL) onto an Aeris PEPTIDE XB-C18 100 × 2.1 mm column, with solid core 1.7 μm particles (100 Å) fitted with a SecurityGuard ULTRA C18-peptide guard column (Phenomenex, Torrance, CA) using a Nexera UHPLC system (Shimadzu, Columbia, MD) with water (A) and ACN (B), both containing 0.1% formic acid, at a flow rate of 0.3 mL/min (40°C). The gradient started at 5% B (held for 2.5 min) and was linearly increased to 30% B within 37.5 min, to 50% B within 10 min, then to 85% B within 5 min (held for 3 min). MS and MS/MS spectra were collected on a high-resolution TripleTOF 5600 system (quadrupole-time-of-flight; Sciex, Concord, ON) equipped with a DuoSpray ion source in positive mode set at 5 kV source voltage, 500°C source temperature, and 50 psi GS1/GS2 gas flows, with a declustering potential of 80 V. The instrument performed a survey MS acquisition (TOFMS) from *m/z* 120–1,250 (250 ms accumulation time), followed by MS/MS (high sensitivity mode) from *m/z* 80–1,500 on the 15 most intense precursor ions (excluded for 20 s after two occurrences) using information dependent acquisition with dynamic background subtraction. Each MS/MS acquisition had an accumulation time of 50 ms and a collision energy (CE) of 30 ±10 V. The total cycle time was 1.05 s. MS and MS/MS calibration was performed after every four injections with a set of in-house standards, using an automatic calibrant delivery system. Sciex Analyst software version 1.7.1 (TripleTOF) was used for data acquisition. Raw data was visualized with PeakView 2.2.

Human files were searched against the UniProtKB/Swiss-Prot protein database (release date: 07/18/2018, including common protein contaminations) by ProteinPilot 5.0 software (Sciex) using the Paragon Algorithm (Shilov et al., 2007). To find potential APAP-related adducts, the feature probability was altered to 50% for NAPQI on cysteine (addition of C_8_H_7_NO_2_). The search was performed for +2 to +4 charge states at a MS tolerance of 0.05 u on precursor ions and 0.1 u on fragment ions. Peptides and proteins were identified with a 1% false discovery rate (FDR) (Tang et al., 2008) using a target-decoy database search algorithm.

### LC-MS/MS Analysis in MRM Mode

LC-MRM experiments were performed on an identical LC platform and column, and a QTRAP 5500 (Sciex, Concord, ON) hybrid quadrupole-linear ion trap system with a TurboIonSpray ion source in positive mode. For fractionated samples, a shorter LC gradient (~28 min) was used. The gradient started at 5% B (held for 2.5 min) and was linearly increased to 30% B within 21.5 min, to 50% B within 2 min, then to 85% B within 0.5 min (held for 2 min). Source parameters were as follows: ion spray voltage, 5 kV; temperature, 550°C; GS1 and GS2, 50 psi; and curtain gas, 35 psi. Entrance and collision cell exit potentials were set at 10 and 13 V, respectively. Ion activation via collision-induced dissociation was performed at collision gas pressure of 5 (arbitrary units). Individual MRM transitions with corresponding CE settings can be found in Table S2. MRM transitions of modified peptides were integrated using Sciex MultiQuant 2.1. Sciex Analyst software version 1.6 was used for data acquisition. Raw data was visualized with PeakView 2.2.

## Results

### Method Development

For unbiased analysis of binding to cysteines in recombinant GSTs, each GST was probed for covalent GSH-cysteine adducts, which could have occurred during GST purification over GSH affinity chromatography. GSTs were digested using trypsin and pepsin without reduction prior to alkylation, and then analyzed by DDA and subsequent database searching. Several cysteine GSH modifications were observed. However, these adducts were found with low absolute abundance compared to free cysteine (alkylated). An average relative signal of 0.03 was estimated, comparing glutathionylated peptides to alkylated peptides.

Optimal *in vitro* metabolism of APAP was examined by GSH trapping experiments (data not shown). Oxidizing RLM and CYP3A4 Supersomes were each tested with either NADPH, or NADP^+^ and a NADPH regenerating system. RLM incubation in combination with a NADPH regenerating system and 3A4 Supersomes in the presence of NADPH yielded optimal conditions for APAP *in vitro* metabolism. For both strategies, a combination of 1 h open tube incubation followed by 2 h closed tube was found best for oxidation. It must be noted that GST stock solutions contained reduced GSH, which also bound to NAPQI (data not shown). Denaturation and excessive buffer exchange of GSTs, to yield completely GSH-free stocks, did not permit the study of binding capabilities based on protein structure. Additionally, excessive treatment of proteins led to important sample loss, different for each protein (data not shown). Furthermore, inclusion of a His-tag during expression (for subsequent Ni-nitrilotriacetic acid purification) might alter protein folding and thus binding results (Ledent et al., 1997). The use of non-mutant GSTs purified over GSH affinity combined with solvent-free elution seemed optimal in this study. These facts were considered and APAP concentrations in final incubations were adjusted accordingly to assure detectable amounts of NAPQI-GST being formed. Subsequently, protein digestion, SPE and offline fractionation were optimized for highest sequence coverage of target GST from database search results of DDA runs. Trypsin and pepsin digestion (4 and 1 h, respectively) in parallel was found optimal to yield a sequence coverage >83% (data not shown). Standard iodo-APAP-GST was used to test cysteine coverage and detection of all possible modified cysteine (see Table 2). Only the sites Cys15 and 102 were detected with a confidence <95%, based on small sequence size and low fragmentation of tryptic C^15^(iodo-APAP)AALR, and poor abundance of peptic GVEDLRC^102^(iodo-APAP)KYISL. For peptide separation, high-pH RP and fraction concatenation was used as an alternative strategy to conventional ion exchange offline fractionation (Zhou, 2003; Yang et al., 2012). This enabled orthogonal separation to online (low-pH) RP chromatography without a desalting SPE step, thus reducing potential sample loss (Wang et al., 2011; Di Palma et al., 2012).

**Table 2:** Coverage of iodo-APAP-cysteine sites in DDA HRMS/MS.

**GST**	**Cys**	**Peptide**	**z**	**Peptide confidence [%]**	**(DDA) absolute precursor area [cps]**	**RT [min]**
**Trypsin digestion**						
M1	78	ITQSNAILC(iodo-APAP)Y	+2	95	6.48E+06	24.4
	87	HNLC(iodo-APAP)GETEEEK	+3	99	1.21E+06	7.0
	115	GMIC(iodo-APAP)YNPEFEK	+2	99	7.99E+05	21.6
	174	C(iodo-APAP)LDAFPNLK	+2	99	1.76E+06	23.8
M2	87	HNLC(iodo-APAP)GESEK	+3	99	9.63E+05	3.4
	115	LC(iodo-APAP)YDPDFEK	+2	95	5.56E+07	20.8
	174	NQVFEPSC(iodo-APAP)LDAFPNLK	+3	99	1.60E+07	32.0
P1	15	C(iodo-APAP)AALR	+2	36	1.92E+07	5.6
	48	ASC(iodo-APAP)LYGQLPK	+2	98	1.11E+07	19.4
**Pepsin digestion**						
A1	112	PVC(iodo-APAP)PPEEKDAKL	+2	99	3.34E+06	14.4
M1	115	GMIC(iodo-APAP)YNPEF	+2	99	6.89E+05	27.9
M2	87	IARKHNLC(iodo-APAP)GESEKEQIRE	+2	99	5.10E+05	7.4
	115	AKLC(iodo-APAP)YDPDF	+2	98	1.28E+07	21.9
	174	ERNQVFEPSC(iodo-APAP)L	+2	99	3.77E+07	23.9
P1	102	GVEDLRC(iodo-APAP)KYISL	+3	32	7.18E+04	23.6
	170	IHEVLAPGC(iodo-APAP)L	+2	99	3.30E+06	23.8

MRM experiments were based on initial DDA results of digested iodo-APAP-GST standards. After database searching, iodo-APAP-modified peptides were investigated for each cysteine site (data not shown). Based on signal abundance and MS/MS properties, up to three modified peptides per cysteine site were chosen (see Table S2); peptide precursor with more than seven residues (Liebler and Zimmerman, 2013) and a charge state of +2 and +3 were preferred. Then, final transitions were based on the three most intense fragment ions (in DDA runs, with *m/z* <1250) with fragment *m/z* > precursor *m/z*. An additional transition was based on the most intense fragment (no *m/z* restriction). CE was further optimized for highest signal abundance of each transition.

### Protein Analysis by DDA and Detection of NAPQI Binding

**Figure 3:**
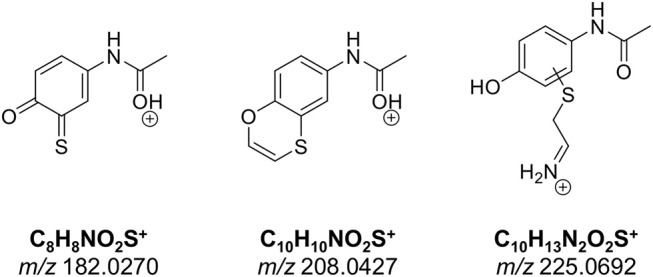
Proposed structures of diagnostic fragment ions.

**Figure 4:**
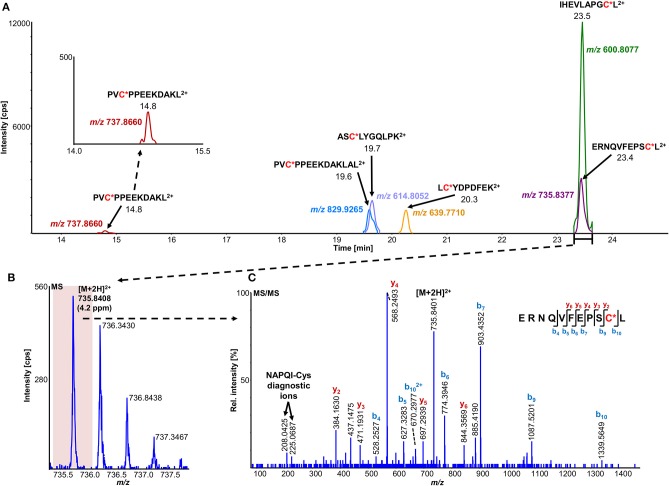
Overlaid high-resolution extracted ion chromatograms (±0.01 u) of detected modified peptide precursors **(A)**. Confirmation was based on high-resolution accurate mass TOFMS **(B)** and triggered (DDA) MS/MS **(C)** analyses, including diagnostic NAPQI-cysteine fragment ions.

The number of found total proteins within 1% FDR ranged from 135 to 356 for RLM incubated, non-fractionated samples; from 40 to 80 for Supersomes incubations. Applying offline fractionation lead to an average increase in found proteins at 1% FDR of 23.7% and 28.5% for RLM and Supersomes incubated samples, respectively. NAPQI-alkylated peptides found by database searching were confirmed by using the following criteria: 1) precursor mass error <10 ppm, 2) MS/MS spectral confidence >95%, 3) the presence of one diagnostic production (see Figure 3) (Sleno et al., 2007; Golizeh et al., 2015), and 4) <0.5 min retention time deviation from iodo-APAP standard (for human GSTs). A total of six NAPQI-modified peptides of five distinct cysteine sites of three GSTs were found based on above criteria (see Table 3). Overlaid extracted ion chromatograms of all six peptides can be found in Figure 4A, Found diagnostic fragment ions can be found in Table 4. Additional MS and MS/MS spectra are available in Figures 4B,C and Figure S1. In addition, NQVFEPSC^*^LDAFPNLK from human GSTM2 and one peptide from rat MGST1 (from RLM background) were identified without detection of diagnostic fragment ions (Figures S2, S3). MS/MS of modified rat MGST1 peptide, VFANPEDC^*^AGFGK, was confirmed by comparison to previously analyzed standard peptide (Golizeh et al., 2015). A comparison of NAPQI-modified peptides from human GSTs to their confidently identified carbamidomethylated variants can be found in Table 5. An overview of all carbamidomethylated GSTs can be found in Table S3.

**Table 3:** NAPQI-GST peptides confirmed by DDA and MRM methods^a^.

**GST**	**Cys**	**Peptide**	**DDA**				**MRM**			
			**RLM (1D)**	**RLM (2D)**	**3A4 (1D)**	**3A4 (2D)**	**RLM (1D)**	**RLM (2D)**	**3A4 (1D)**	**3A4 (2D)**
A1	112	PVC*PPEEKDAKL		✓			✓	✓		✓
		PVC*PPEEKDAKLAL	✓				✓	✓		
M1	78	ITQSNAILC*Y					✓		✓	✓
M2	115	AKLC*YDPDF						✓		✓
		LC*YDPDFEK		✓						
	174	ERNQVFEPSC*L				✓	✓	✓	✓	✓
		DVLERNQVFEPSC*								✓
		NQVFEPSC*LDAFPNLK	✓	✓			✓	✓	✓	✓
P1	15	C*AALR								✓
	48	ASC*LYGQLPK				✓				✓
	170	IHEVLAPGC*L	✓				✓	✓	✓	✓

a *RLM (1D): RLM incubation without fractionation, RLM (2D): RLM incubation with fractionation, 3A4 (1D): CYP3A4 Supersome incubation without fractionation, 3A4 (2D): CYP3A4 Supersome incubation with fractionation, DDA: confirmed by DDA, MRM: confirmed by MRM*.

**Table 4:** NAPQI-cysteine diagnostic fragment ions (MS2) detected in HRMS/MS experiments.

**GST**	**Cys**	**Peptide**	**Diagnostic ion (ppm)**		
			***m/z* 182.0270**	***m/z* 208.0427**	***m/z* 225.0692**
A1	112	PVC*PPEEKDAKL		✓ (−2.4)	✓ (−0.4)
		PVC*PPEEKDAKLAL		✓ (−1.9)	✓ (−1.8)
M2	115	LC*YDPDFEK			✓ (1.8)
	174	ERNQVFEPSC*L		✓ (−1.0)	✓ (−2.2)
P1	48	ASC*LYGQLPK	✓ (5.5)		✓ (5.8)
	170	IHEVLAPGC*L		✓ (−9.6)	

**Table 5:** Comparison of NAPQI-peptide to the IAM-modified (CAM) version found with >95% confidence in the same DDA experiments.

**GST**	**Cys**	**Peptide**	**NAPQI modification**		**CAM modification**	
			**RT [min]**	**Absolute peptide intensity [cps] (z)**	**RT [min]**	**Absolute peptide intensity [cps] (z)**
A1	112	PVC*PPEEKDAKL	14.8	9.96E+02 (+3)	12.1	8.63E+03 (+3)
		PVC*PPEEKDAKLAL	19.6	6.61E+03 (+3)	17.1	1.77E+04 (+2)
M2	115	LC*YDPDFEK	20.3	3.83E+02 (+2)	17.2	5.86E+04 (+2)
	174	ERNQVFEPSC*L	23.4	1.27E+03 (+2)	19.3	3.19E+05 (+2)
		NQVFEPSC*LDAFPNLK	31.6	1.84E+03 (+2)	28.9	2.68E+05 (+2)
P1	48	ASC*LYGQLPK	19.7	5.82E+02 (+2)	15.5	1.09E+04 (+2)
	170	IHEVLAPGC*L	23.5	3.87E+03 (+2)	19.5	1.00E+02 (+1)

### Confirmation of NAPQI Binding by MRM Strategies

NAPQI-modified peptides were identified in MRM experiments by following criteria: 1) signal-to-noise (S/N) of all transitions >10, 2) <17.2% deviation of relative abundance (see Equation 1) (Loziuk et al., 2014) of two highest transitions (with fragment *m/z* > precursor *m/z*) from iodo-APAP-modified peptide standard, and 3) <0.5 min retention time deviation of all transitions from iodo-APAP standard. After data filtration, MRM strategies yielded 10 NAPQI-modified peptides of seven cysteine sites of four GSTs (see Table 3). Overlaid MRM chromatograms can be found in Figures S4–S7. The relative abundance of transitions was calculated through the ratio of its integrated peak area and the peak area sum of all three transitions (with fragment *m/z* > precursor *m/z*) (Loziuk et al., 2014):

(1) $$\begin{array}{r} \text{Relative abundance}=\frac{{\text{A}}_{\text{x}}}{\sum_{i=1}^{3}{\text{A}}_{i}} \end{array}$$

### Comparison of Different Strategies

**Figure 5:**
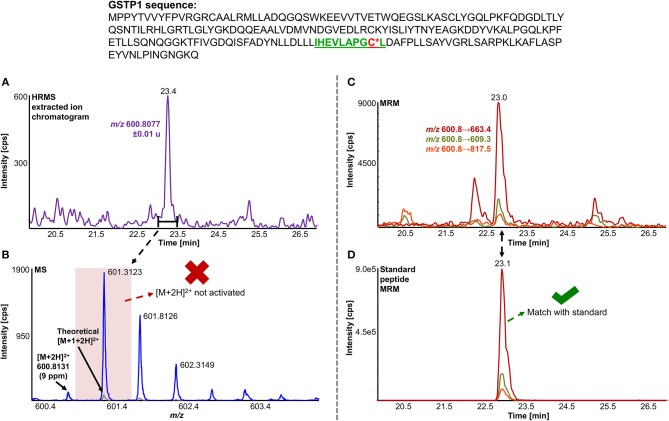
Comparison of LC-DDA and LC-MRM results, used to identify NAPQI-modified GSTP1 peptide IHEVLAPGC^170^L (underlined) from *in vitro* incubation with CYP3A4 (Supersomes). DDA analysis of the target peptide (theoretical [M+2H]^2+^ at *m/z* 600.8077) eluting at 23.4 min **(A)** did not yield MS/MS acquisition **(B)**. Fragmentation was triggered by an interfering ion at *m/z* 601.3123, which resulted in a MS1 isolation window (red rectangle) not incorporating target peptide. MRM analysis **(C)** confirmed presence of IHEVLAPGC*L^2+^ by comparing to iodo-APAP-GST **(D)**.

A total of 11 distinct peptides comprising seven unique cysteine sites were found as NAPQI-modified, two of which, Cys78 in GSTM1 and Cys15 in GSTP1, were uniquely found by MRM strategies. Thus, CYP3A4 incubations combined with high-pH RP fractionation and MRM analysis was found most suitable with a total of nine found distinct peptides, including all seven distinct cysteine sites. This combination reflects the least biological background, reduced matrix effects by offline fractionation and more certain detection by following selective transitions at matching retention times of peptides modified by the isomeric labeling reagent. Peptides ERNQVFEPSC^*^L, NQVFEPSC^*^LDAFPNLK (both Cys174 in GSTM2), and IHEVLAPGC^*^L (Cys170 in GSTP1) were identified using all four tested MRM strategies. Using MRM techniques demonstrated that dilution effects during offline fractionation, in the case where a specific peptide can elute over neighboring fractions (see Figure S8), could lead to target signal intensities below detection limits (S/N <10) and thus missing identification in DDA and MRM experiments. This was observed in RLM incubations of GSTM1. Peptide ITQSNAILC^*^Y was only detected in MRM experiments without LC fractionation (see Table 3). Furthermore, DDA experiments showed higher susceptibility to interfering compounds, even when detection limits were not an issue. Co-eluting, high abundant interferences in TOFMS survey scans resulted in missed MS/MS acquisition of target peptides. A visual representation of missed fragmentation of a NAPQI-peptide target (IHEVLAPGC^*^L), compared to a positive MRM identification, can be found in Figure 5. Missing MS/MS acquisition of the IHEVLAPGC^*^L peptide precursor at *m/z* 600.8077 was caused by a higher abundant interference at *m/z* 601.3123, overlapping with the peptide [M+1+2H]^2+^ signal. In that case, only the highly abundant interference was analyzed in MS2 leading to a MS/MS spectrum of interference fragments and low abundant [M+1+2H]^2+^ fragments, not allowing confident identification. One peptide, LC^*^YDPDFEK (Cys115 in GSTM2), was found only through DDA efforts, which was based on susceptibility of MRM transitions to isobaric interferences. The peptide was ultimately identified through HRMS/MS sequencing only (see Figure S1C). These circumstances lead to differences between DDA or MRM analyses (e.g., RLM (1D) compared to 3A4 (1D) in Table 3), leading to more found modified sites with RLM incubations than with Supersomes, even though the latter were less complex incubations. Overall, NAPQI-peptide MRM signals reached higher signal abundance as extracted precursor ion (±0.01 u) signals in DDA survey scans (TOFMS) with comparable S/N. The use of both trypsin and pepsin as proteases was shown to be complementary. Several sites were uniquely found using either one of the two proteases. NAPQI-modified Cys15, 48, and 78 were uniquely found in trypsin digests, whereas Cys112 and 170 were only found after pepsin digestion. Cys115 and 174 in GSTM2 were the only sites with detectable NAPQI peptides from both protease treatments; AKLC^*^YDPDF and LC^*^YDPDFEK, and ERNQVFEPSC^*^L and NQVFEPSC^*^LDAFPNLK, respectively.

## Discussion

The GST family is an essential, multifunctional set of enzymes, crucial in many detoxification and binding reactions in liver and other tissues. GSTs are involved in the metabolism of many substances and xenobiotics, including reactive metabolites such as NAPQI. The understanding of NAPQI binding to GSTs is thus a logical step toward understanding its hepatotoxicity. This study used several approaches for targeted and untargeted identification of modified peptides from *in vitro* incubations. The use of different methodologies yielded complementary findings but also allowed a critical view on performance and inherent limitations of each method.

### Performance of Different Strategies

This study highlighted the importance of using complementary proteases to better cover potential modification sites. Pre-fractionation of peptides was mostly beneficial for identifying adducted peptides. The double protease approach substantially increased protein sequence coverage. Multiple protease digestion can be easily applied in proteomics workflows and is highly flexible, due to wide range of specific and less specific enzymes with diverse cleavage behavior. Pre-fractionation increased peptide coverage and improved NAPQI-peptide discovery due to better resolution of peptides, thus reducing matrix effects in both MRM and DDA. Benefits from better peptide resolution in multiplexed LC outweighed the potential for sample loss and dilution effects introduced through fractionation and concatenation. These sample preparation techniques are viable approaches to broaden the potential for identification of low abundant modifications on proteins.

Targeted MRM-based methods are most easily developed with the availability of purified proteins, whereas untargeted DDA analyses offer a more flexible, universal application without time-consuming optimization steps. GSTs are relatively easy to purify via GSH affinity chromatography and thus represent ideal proteins for reference standard preparation to build and optimize MRM methods. Untargeted DDA with subsequent database searching did not require any custom-made standards. Furthermore, DDA allowed for the analysis of protein binding beyond recombinant GST as the only target during incubations. For example, database searching for NAPQI-modified RLM proteins detected modified rat MGST1. Moreover, DDA and MRM strategies did not only differ in terms of applicability but also performance. This was rooted in differences in both MS platforms, as well as the two different acquisition types. Low resolving triple quadrupole systems (here a quadrupole-linear ion trap hybrid) generally reach lower limits of detection based on a higher duty cycle in optimized MRM modes. In comparison, quadrupole-time-of-flight instruments are less sensitive (Geib et al., 2016) but offer higher mass accuracy and the possibility for structural characterization by MS/MS. The difference in sensitivity is a crucial factor in characterizing low abundant protein modifications. In our findings, MRM strategies generally reached superior detection limits and thus led to two uniquely found modified peptides. In addition, even when detection limits were surpassed, low abundance of NAPQI-peptides resulted in missed peptide precursor fragmentation in DDA and thus were not identified. The final number of found modified sites, confirmed by DDA, was ultimately achieved here by using multiple attempts, which highlighted the limitations of unsupervised DDA in covalent adduct analysis. Furthermore, DDA relies on automatically triggered high quality MS/MS of modified peptides. This can be a challenge in complex biological samples, especially for low-abundant modifications, since so many peptides are eluting off the analytical column together. Even though high-speed high-resolution mass spectrometers, such as the one used in this work, have the ability to acquire spectra very fast and with high sensitivity, there are still many peptides not triggered for MS/MS acquisition under these conditions. MRM, on the other hand, does not suffer from this caveat. If a given peptide is present, above a certain threshold, it should be detected by MRM. This method still suffers from matrix effects (ion suppression) but is not competing with co-eluting species for MS/MS acquisition. In general, MRM methodologies offer higher robustness and reproducibility. However, it should be noted that the use of a semi-targeted DDA, using an inclusion list of putative modified peptides could potentially increase reproducibility. Furthermore, susceptibility of low mass resolving MRM to isobaric interferences might obstruct confident identification and quantitation in some cases, which is less likely in HRMS. Also constant improvements in instrumentation and software are improving DDA performances and the development of all-ion fragmentation in data-independent acquisition (Bruderer et al., 2015) will certainly be viable avenues for future applications of HRMS in protein binding studies. In conclusion, MRM and DDA are powerful MS tools in proteomics, both of which have intrinsic advantages and disadvantages.

### Putative NAPQI-cysteine Sites in GST

In this study we characterized seven cysteine sites from four cytosolic human GSTs by NAPQI *in vitro*. NAPQI binding to human GSTA1, M1, and M2 had not been reported previously. Only binding to Cys48 and 102 in human GSTP1 was previously confirmed (Jenkins et al., 2008; Boerma et al., 2011). In one of their studies, Boerma et al. (2011) also reported binding of clozapine metabolites to Cys48, as well as metabolized troglitazone to Cys15, 48, and 102 by *in vitro* incubation, tryptic digestion, affinity removal of background protein and LC-MS/MS analysis. In a follow-up study Boerma et al. (2011) confirmed also diclofenac-related binding to Cys15 and 48. Using GSTP1 in a protein trapping study, binding of the reactive diquinone methide of raloxifene was also observed to Cys48 (Yukinaga et al., 2012). Here, we detected modified Cys48 and additional target sites of NAPQI in GSTP1 by using pepsin as an additional protease with different cleavage specificity than trypsin. We observed Cys15, 48, and 170 as NAPQI target sites in GSTP1. Boerma et al. (2011) stated that alkylation of Cys48 lead to complete inhibition of GSTP1, based on the close proximity to GSH binding sites (Reinemer et al., 1991; Vega et al., 1998), as well as subsequent disruption of the active GSTP1-1 dimer (Jenkins et al., 2008). Additionally, modification on Cys170 is believed to influence activity as well (Orton and Liebler, 2007), which is also explained by its importance in dimerization (Chang et al., 2001). Furthermore, Cys15 is in direct proximity to Arg14 which is also involved in GSH binding (Ji et al., 1997; Oakley et al., 1997a,b; Prade et al., 1997; Ang et al., 2009; Federici et al., 2009). It is possible that alkylation of multiple sites combined leads to full enzymatic inhibition. Binding analysis of Cys102 presented several challenges in this study. The site was inaccessible via tryptic digestion and peptic peptides showed the lowest signal intensity of all screened sites. Sufficient recovery of Cys102 requires other proteases or potentially more limited proteolysis. Jenkins et al. (2008) used Asp-N to confirm binding of NAPQI to Cys102. However, it was reported that Cys102 binding occurred only with high molar ratios of NAPQI to protein. Alkylation reactions of Cys102, being more solvent exposed, was believed to play a minor role in enzymatic activity (Lemercier et al., 2004).

Previous alkylation studies of GSTA1 focused on *in vivo* binding of bromobenzene metabolites to Cys112 in rat (Koen et al., 2006). Detection of these low abundant peptide modifications was enabled by GSH affinity purification, tryptic digestion and capillary LC-MS. Human and rat GSTA1 share a high identity of 76%, including Cys112, which was found NAPQI-modified in our work. The relatively long distance between Cys112 and the active sites, Tyr9 and Arg45 (Balogh et al., 2009), was believed to ensure activity even after cysteine alkylation (Koen et al., 2006). However, Cys112 is in range to Met94, Phe136, and Val139, which are associated to a hydrophobic pocket, important in dimerization (Vargo et al., 2004). Alkylation of Cys112 could therefore compromise the formation of the GSTA1-1 dimer. However, further activity studies of NAPQI-modified GSTA1 are needed to investigate this.

In comparison to our findings of NAPQI-modified Cys78 in GSTM1, Nerland et al. (2001) found rat GSTM1 (Cys87) modified *in vivo* by acrylonitrile using a combination of radiolabeling, affinity purification and LC-MS. Modification of Cys87 in rat was also observed by a recent study of Leeming et al. (2017), using *in vitro* metabolism of APAP and LC-MS/MS. Rat GSTM1 is missing Cys78, though Cys87 is identical in human and rat. Hence, an interspecies comparison of cysteine reactivity is not trivial, since both sites, Cys78 and 87, might compete for binding reactions. However, Nerland et al. (2001) hypothesized that the reactivity of Cys87 is based on possible interactions with His85, which is only found in rat. Missing His85 could explain a lower reactivity of Cys87 in human GSTM1 (and M2). Nevertheless, the extent of enzyme inhibition after modification needs further study. Nerland et al. (2001) observed loss in rat GSTM1 activity only after IAM alkylation of Cys115 (identical in human GSTM1) and not Cys87. A possible explanation might be the close active site Tyr116, which is vital for substrate binding (Johnson et al., 1993). Binding to Cys115 in human GSTM1 was not observed in our experiments. However, we could identify Cys115 and 174 as putative targets in human GSTM2. Koen et al. (2012) studied *in vivo* binding of 4-bromophenol in rat GSTM2 and confirmed adducts by radioactive labeling, tryptic digestion and LC-MS/MS. Based on cysteine mutant studies of human GSTM2, it has been stated that Cys115 might be involved in enzyme activity (Norrgård et al., 2011), based on the mu class active site Tyr116 (Johnson et al., 1993). The effect of binding to GSTM2 needs further research, especially with regards to distinguishing both modified sites found in this study, Cys115 and 174. Furthermore, the difference of NAPQI binding properties of GSTM1 and GSTM2 are also crucial to investigate in future studies, since they share very high sequence homology.

In conclusion, we used *in vitro* incubations to identify adduct formation with NAPQI. Further research of *in vivo* and *in vitro* treated hepatocytes and liver fractions would complement our findings. The techniques shown here were designed to help study protein adduct formation and to guide subsequent research. Furthermore, a combination of our methods with targeted sample preparation and enrichment tools (e.g., immunoaffinity) could be applied to analyze the NAPQI binding affinity of GST and various proteins of interest in the future. A similar strategy can be used to find NAPQI-protein targets in more complex samples and unknown protein targets, while pinpointing binding sites. The novel use of an isomeric labeling reagent with identical chromatographic elution of NAPQI-modified cysteine containing peptides is invaluable for confirming the identity of modified peptides.

## Data Availability

The datasets generated for this study are available on request to the corresponding author.

## Author Contributions

LS and TG conceived the research. CL and DW performed recombinant expression and purification of GST proteins. TG carried out further sample preparation, experiments, analyses, and data treatment. TG and LS wrote the paper. All authors made substantial, direct and intellectual contribution to the work, and revised the manuscript.

### Conflict of Interest Statement

The authors declare that the research was conducted in the absence of any commercial or financial relationships that could be construed as a potential conflict of interest.

## Supplementary Material

Additional information on protein detection methods, MRM transitions and identified carbamidomethylated GST peptides; figures of peptide chromatograms, MS and MS/MS spectra, and peptide distribution in concatenated fractions.
