# Supplementary material for: Liquid Chromatography-Tandem Mass Spectrometry Analysis of Acetaminophen Covalent Binding to Glutathione S-Transferases
Source: Front Chem. 2019 Aug 13;7:558. doi: 10.3389/fchem.2019.00558 (PMC6700392; doi:10.3389/fchem.2019.00558)
Supplement: Supplementary file 1 [file Data_Sheet_1.docx]

**Supporting Information**

**Liquid Chromatography-Tandem Mass Spectrometry Analysis of Acetaminophen Covalent Binding to Glutathione *S*-Transferases**

**Running Title: LC-MS/MS Analysis of NAPQI-GST**

Timon Geib^1^, Cristina Lento^2^, Derek J. Wilson^2^, and Lekha Sleno^1^*

*^1^Chemistry Department, Université du Québec à Montréal, Montréal, QC, Canada*

*^2^Department of Chemistry/The Centre for Research in Mass Spectrometry, York University, Toronto, ON, Canada*

***Correspondence:**

Lekha Sleno, sleno.lekha@uqam.ca

**Table of Content:**

Table S1 A brief comparison of protein detection methods. 3

Table S2 List of MRM transitions for targeted screening of modified peptides. 4

Table S3 List of identified (peptide confidence >95%) carbamidomethylated (CAM)

cysteines in GSTA1, M1, M2 and P1. 11

Figure S1A DDA spectra of NAPQI-GSTA1 peptic peptide PVC*PPEEKDAKL

(acquired at 14.8 min). 18

Figure S1B DDA spectra of NAPQI-GSTA1 peptic peptide PVC*PPEEKDAKLAL

(acquired at 19.6 min). 19

Figure S1C DDA spectra of NAPQI-GSTM2 tryptic peptide LC*YDPDFEK

(acquired at 20.3 min). 20

Figure S1D DDA spectra of NAPQI-GSTP1 tryptic peptide ASC*LYGQLPK

(acquired at 19.7 min). 21

Figure S1E DDA spectra of NAPQI-GSTP1 peptic peptide IHEVLAPGC*L

(acquired at 23.5 min). 22

Figure S2 Extracted ion chromatogram of NAPQI-GSTM2 tryptic peptide NQVFEPSC*LDAFPNLK (A), including survey scan MS (B) and triggered MS/MS (C). 23

Figure S3 DDA spectra of NAPQI-rat MGST1 tryptic peptide VFANPEDC*AGFGK

(acquired at 22.0 min). 24

Figure S4 Overlaid MRM chromatograms (60 min LC) of found modified peptides

using RLM incubation and SPE purification. 25

Figure S5 Overlaid MRM chromatograms (30 min LC) of found modified peptides

using RLM incubation, SPE purification and high-pH RP fractionation. 26

Figure S6 Overlaid MRM chromatograms (60 min LC) of found modified peptides

using CYP-3A4 Supersomes incubation and SPE purification (A), including peptide ITQSNAILC*Y (B). 27

Figure S7 Overlaid MRM chromatograms (30 min LC) of found modified peptides

using CYP3A4 Supersomes incubation, SPE purification and high-pH RP fractionation (A), including peptide C*AALR (B). 28

Figure S8 Distribution of selected MRM transitions over all concatenated fractions of peptides NQVFEPSC*LDAFPNLK (A), C*AALR (B) and PVC*PPEEKDAKL (C), showing individual differences in elution during high-pH RP fractionation. 29

**Table S1** A brief comparison of protein detection methods.

| **Method** | **Detection** | **Possible quantitation** | **Representative reference** |
| --- | --- | --- | --- |
| Bottom-up proteomics LC-MS/MS | Peptide sequencing with MS/MS fragmentation | Stable isotopic labeling (or label-free) | Cao, Z. et al. (2017) *Plant Methods* 13, 64. |
| Fluorescent probe | Fluorophore-labeled proteins | Fluorescence signal | He, N. et al. (2019) *Anal. Chem.* 91, 5424–5432. |
| Radiolabeling | Autoradiography of labeled proteins | Liquid scintillation counting | Nerland, D. E. et al. (2001) *Chem. Res. Toxicol.* 14, 799–806. |
| Enzyme-linked immunosorbent assay | Direct or indirect detection of conjugated enzyme activity | Spectrophotometry | Tsuchida, S. et al. (1989) *Cancer Res.* 49, 5225–5229. |
| Western blotting | Colorimetric, chemiluminescent or fluorescence detection | Through spectrophotometry | Howie, A. F. et al. (1990) *Carcinogenesis* 11, 451–458. |

**Table S2** List of MRM transitions for targeted screening of modified peptides.

| **GST** | **Site** | **Peptide precursor ion** | **Fragment ion: transition (CE [V])** |
| --- | --- | --- | --- |
|  |  | **Trypsin digestion** |  |
| A1 | Cys112 | LLPVC*PPEEK^2+^ | y_6_: *m/z* 637.3→851.4 (32), y_8_: *m/z* 637.3→1047.5 (27), b_5_: *m/z* 637.3→675.4 (22) and y_8_^2+^: *m/z* 637.3→524.2 (27) |
|  |  | ILLLPVC*PPEEK^2^ | y_8_: *m/z* 750.4→1047.5 (28), y_9_: *m/z* 750.4→1160.6 (28), y_6_: *m/z* 750.4→851.4 (32) and b_3_: *m/z* 750.4→340.3 (32) |
|  |  | ADLGEMILLLPVC*PPEEK^2^ | y_9_: *m/z* 1058.5→1160.9 (32), b_10_: *m/z* 1058.5→1069.6 (30), b_11_: *m/z* 1058.5→1166.6 (32) and b_9_: *m/z* 1058.5→956.5 (32) |
| M1 | Cys78 | ITQSNAILC*Y^2+^ | b_7_: *m/z* 637.8→728.4 (18), b_8_: *m/z* 637.8→841.5 (18), b_9_: *m/z* 637.8→1093.5 (18) and y_2_: *m/z* 637.8→434.1 (23) |
|  |  | ITQSNAILC*YIAR^3+^ | y_5_: *m/z* 539.0→774.4 (23), y_6_: *m/z* 539.0→887.4 (18), b_6_: *m/z* 539.0→615.3 (22) and y_4_: *m/z* 539.0→522.3 (27) |
|  |  | ITQSNAILC*Y^3+^ | y_2_: *m/z* 425.5→434.1 (23), b_5_: *m/z* 425.5→544.3 (18), b_6_: *m/z* 425.5→615.3 (18) and C*: *m/z* 425.5→225.1 (27) |
|  | Cys87 | KHNLC*GETEEEKIR^3+^ | y_5_: *m/z* 612.3→674.4 (32), y_7_: *m/z* 612.3→904.5 (32), y_13_^2+^: *m/z* 612.3→853.9 (30) and y_13_^3+^: *m/z* 612.3→569.6 (28) |
|  |  | HNLC*GETEEEK^3+^ | y_4_: *m/z* 479.9→534.2 (18), y_5_: *m/z* 479.9→635.3 (18), y_7_: *m/z* 479.9→821.4 (18) and b_2_: *m/z* 479.9→252.1 (32) |
|  |  | HNLC*GETEEEKIR^3+^ | y_5_: *m/z* 569.6→674.4 (32), y_6_: *m/z* 569.6→803.4 (28), y_7_: *m/z* 569.6→904.5 (28) and b_2_: *m/z* 569.6→252.1 (32) |

**Table S2** Continued.

| **GST** | **Site** | **Peptide precursor ion** | **Fragment ion: transition (CE [V])** |
| --- | --- | --- | --- |
|  |  | **Trypsin digestion** |  |
| M1 | Cys115 | MIC*YNPEFEK^2+^ | y_6_: *m/z* 711.8→763.4 (28), y_7_: *m/z* 711.8→926.4 (28), y_8_: *m/z* 711.8→1178.5 (28) and a_2_: *m/z* 711.8→217.1 (32) |
|  |  | GMIC*YNPEFEK^2+^ | y_6_: *m/z* 740.3→763.4 (28), y_7_: *m/z* 740.3→926.4 (32), y_8_: *m/z* 740.3→1178.5 (28) and y_5_: *m/z* 740.3→649.3 (32) |
|  |  | C*YNPEFEK^2+^ | y_5_: *m/z* 589.7→649.3 (28), y_6_: *m/z* 589.7→763.4 (27), y_7_: *m/z* 589.7→926.4 (27) and b_2_: *m/z* 589.7→416.1 (23) |
|  | Cys174 | C*LDAFPNLK^2+^ | y_5_: *m/z* 585.3→618.4 (23), y_6_: *m/z* 585.3→689.4 (27), y_7_: *m/z* 585.3→804.4 (27) and y_4_: *m/z* 585.3→471.3 (32) |
|  |  | C*LDAFPNLKDFISR^3+^ | y_6_: *m/z* 596.6→765.4 (32), y_9_: *m/z* 596.6→1089.6 (28), y_12_^2+^: *m/z* 596.6→711.9 (25) and a_2_: *m/z* 596.6→338.2 (32) |
|  |  | C*LDAFPNLKDFISRFEGLEK^3+^ | y_12_^2+^: *m/z* 623.6→734.9 (30), y_15_^2+^: *m/z* 623.6→897.0 (28), y_18_^2+^: *m/z* 623.6→709.4 (23) and a_2_: *m/z* 623.6→338.2 (32) |
| M2 | Cys87 | HNLC*GESEK^3+^ | y_4_−H_2_O: *m/z* 389.2→474.2 (22), y_4_: *m/z* 389.2→492.3 (18), y_5_: *m/z* 389.2→549.3 (18) and b_2_: *m/z* 389.2→252.1 (27) |
|  |  | KHNLC*GESEKEQIR^3+^ | y_5_: *m/z* 607.3→673.4 (32), y_9_: *m/z* 607.3→1075.5 (32), y_13_^2+^: *m/z* 607.3→846.4 (25) and y_13_^3+^: *m/z* 607.3→564.6 (27) |
|  |  | HNLC*GESEKEQIR^3+^ | y_5_: *m/z* 564.6→673.4 (30), y_7_: *m/z* 564.6→889.5 (30), y_12_^2+^: *m/z* 564.6→777.9 (25) and y_4_: *m/z* 564.6→545.3 (20) |

**Table S2** Continued.

| **GST** | **Site** | **Peptide precursor ion** | **Fragment ion: transition (CE [V])** |
| --- | --- | --- | --- |
|  |  | **Trypsin digestion** |  |
| M2 | Cys115 | LC*YDPDFEK^2+^ | y_6_: *m/z* 639.8→750.3 (28), y_7_: *m/z* 639.8→913.43 (25), y_8_: *m/z* 639.8→1165.5 (28) and b_2_: *m/z* 639.8→366.1 (25) |
|  |  | LC*YDPDFEKLKPEYL^3+^ | y_12_^2+^: *m/z* 674.7→689.9 (30), y_13_^2+^: *m/z* 674.7→828.9 (27), b_14_^2+^: *m/z* 674.7→945.9 (22) and y_4_: *m/z* 674.7→521.3 (32) |
|  |  | C*YDPDFEK^2+^ | y_5_: *m/z* 583.2→635.3 (30), y_6_: *m/z* 583.2→750.3 (25), y_7_: *m/z* 583.2→913.4 (25) and a_2_: *m/z* 583.2→388.1 (30) |
|  | Cys174 | NQVFEPSC*LDAFPNLK^3+^ | y_7_: *m/z* 657.6→804.4 (25), y_8_: *m/z* 657.6→917.5 (25), b_8_: *m/z* 657.6→1054.4 (20) and y_4_: *m/z* 657.6→471.3 (20) |
|  |  | C*LDAFPNLKDFISR^3+^ | y_6_: *m/z* 596.6→765.4 (32), y_9_: *m/z* 596.6→1089.6 (30), y_12_^2+^: *m/z* 596.6→711.9 (25) and a_2_: *m/z* 596.6→338.2 (30) |
|  |  | EPSC*LDAFPNLK^2+^ | y_7_: *m/z* 741.9→804.4 (32), y_8_: *m/z* 741.9→917.5 (32), b_8_−H_2_O: *m/z* 741.9→994.4 (22) and y_4_: m/z 741.9→471.3 (32) |
| P1 | Cys15 | C*AALR^2+^ | y_3_: m/z 341.7→359.2 (18), y_4_: *m/z* 341.7→430.3 (20), b_3_: *m/z* 341.7→395.1 (18) and y_1_: *m/z* 341.7→175.1 (28) |
|  | Cys48 | ASC*LYGQLPK^2+^ | y_6_: m/z 614.8→705.4 (28), y_7_: m/z 614.8→818.5 (28), y_8_: *m/z* 614.8→1070.5 (27) and y_5_: *m/z* 614.8→542.3 (27) |
|  |  | C*LYGQLPK^2+^ | y_5_: *m/z* 535.8→542.3 (27), y_6_: *m/z* 535.8→705.4 (23), y_7_: *m/z* 535.8→818.5 (23) and a_2_: *m/z* 535.8→338.2 (28) |

**Table S2** Continued.

| **GST** | **Site** | **Peptide precursor ion** | **Fragment ion: transition (CE [V])** |
| --- | --- | --- | --- |
|  |  | **Trypsin digestion** |  |
| P1 | Cys48 | ASC*LYGQLPK^3+^ | y_4_−NH_3_: *m/z* 410.2→468.3 (22), y_5_: *m/z* 410.2→542.3 (18), b_4_: *m/z* 410.2→524.2 (18) and y_2_: *m/z* 410.2→244.2 (28) |
|  | Cys102 | TLGLYGKDQQEAALVDMVNDGVEDLRC*K^4+^ | y_13_^2+^: *m/z* 808.1→821.9 (28), y_14_^2+^: *m/z* 808.1→871.9 (27), y_26_^3+^: *m/z* 808.1→1005.8 (32) and b_2_: *m/z* 808.1→215.1 (32) |
|  | Cys170 | IHEVLAPGC*LDAFPLLSAY^3+^ | b_15_^2+^: *m/z* 726.7→863.4 (28), b_16_^2+^: *m/z* 726.7→920.0 (27), b_18_^2+^: *m/z* 726.7→999.0 (32) and y_2_: *m/z* 726.7→253.1 (32) |
|  |  | LIHEVLAPGC*LDAFPLLSAY^3+^ | b_7_: *m/z* 764.4→776.5 (32), b_17_^2+^: *m/z* 764.4→976.5 (22), b_19_^2+^: *m/z* 764.4→1055.6 (18) and y_2_: *m/z* 764.4→253.1 (27) |
|  |  | **Pepsin digestion** |  |
| A1 | Cys112 | PVC*PPEEKD^2+^ | y_5_: *m/z* 581.8→617.3 (32), y_6_: *m/z* 581.8→714.3 (30), y_7_: *m/z* 581.8→966.4 (25) and a_3_: *m/z* 581.8→421.2 (30) |
|  |  | PVC*PPEEKDAKL^2+^ | y_8_: *m/z* 737.9→929.5 (30), y_9_: *m/z* 737.9→1026.5 (30), b_9_: *m/z* 737.9→1144.5 (30) and b_3_: *m/z* 737.9→449.2 (32) |
|  |  | PVC*PPEEKDAKLAL^3+^ | y_11_: *m/z* 553.6→1210.7 (25), y_11_^2+^: *m/z* 553.6→605.8 (25), y_12_^2+^: *m/z* 553.6→731.9 (22) and b_3_: *m/z* 553.6→449.2 (30) |
| M1 | Cys87 | IARKHNLC*GETEE^3+^ | (b_11_−H_2_O)^2+^: *m/z* 550.3→677.8 (22), b_11_^2+^: *m/z* 550.3→686.8 (23), b_12_^2+^: *m/z* 550.3→751.4 (23) and y_2_−H_2_O: *m/z* 550.3→241.1 (27) |
|  |  | YIARKHNLC*GETEE^3+^ | b_12_^2+^: *m/z* 647.6→768.4 (22), b_13_^2+^: *m/z* 647.6→832.9 (22), b_14_^2+^: *m/z* 647.6→897.4 (22) and y_2_−H_2_O: *m/z* 647.6→259.1 (32) |

**Table S2** Continued.

| **GST** | **Site** | **Peptide precursor ion** | **Fragment ion: transition (CE [V])** |
| --- | --- | --- | --- |
|  |  | **Pepsin digestion** |  |
| M1 | Cys87 | IARKHNLC*GETEE^2+^ | y_8_: *m/z* 824.9→1043.4 (25), y_9_: *m/z* 824.9→1180.5 (25), b_8_: *m/z* 824.9→1085.6 (25) and y_12_^2+^: *m/z* 824.9→751.4 (25) |
|  | Cys115 | GMIC*YNPEF^2+^ | b_5_: *m/z* 611.7→717.3 (28), b_6_: *m/z* 611.7→831.3 (18), b_8_: *m/z* 611.7→1057.4 (23) and y_3_: *m/z* 611.7→392.2 (28) |
|  |  | MIC*YNPEF^2+^ | b_4_: *m/z* 583.2→660.3 (18), b_5_: *m/z* 583.2→774.3 (22), b_7_: *m/z* 583.2→1000.4 (23) and b_3_: *m/z* 583.2→497.2 (28) |
|  |  | IRVDILENQTMDNHMQLGMIC*YNPEFEK^4+^ | y_8_: *m/z* 883.4→1178.5 (28), b_18_^2+^: *m/z* 883.4→1055.0 (30), b_19_^2+^: *m/z* 883.4→1120.5 (30) and y_5_: *m/z* 883.4→649.3 (30) |
| M2 | Cys87 | IARKHNLC*GESEKEQIRE^4+^ | y_5_: *m/z* 573.0→674.3 (30), y_6_: *m/z* 573.0→802.4 (30), b_12_^2+^: *m/z* 573.0→744.4 (25) and y_4_: *m/z* 573.0→545.3 (30) |
|  |  | RYIARKHNLC*GESEKEQIRE^4+^ | y_5_: *m/z* 652.8→674.3 (32), y_6_: *m/z* 652.8→802.4 (32), b_12_^2+^: *m/z* 652.8→795.9 (32) and y_4_: *m/z* 652.8→545.3 (32) |
|  |  | IARKHNLC*GESEKEQIRE^3+^ | y_7_: *m/z* 763.7→931.5 (32), y_8_: *m/z* 763.7→1018.5 (32) and y_10_: *m/z* 763.7→1204.6 (32) |
|  | Cys115 | AKLC*YDPDF^2+^ | b_5_: *m/z* 610.8→728.3 (20), b_6_: *m/z* 610.8→843.4 (25), b_8_: *m/z* 610.8→1055.5 (25) and y_3_: *m/z* 610.8→378.2 (25) |
|  |  | AKLC*YDPDFE^2+^ | b_6_: *m/z* 675.3→843.4 (25), b_8_: *m/z* 675.3→1055.5 (27), b_9_: *m/z* 675.3→1202.5 (25) and y_4_: *m/z* 675.3→507.2 (25) |

**Table S2** Continued.

| **GST** | **Site** | **Peptide precursor ion** | **Fragment ion: transition (CE [V])** |
| --- | --- | --- | --- |
|  |  | **Pepsin digestion** |  |
| M2 | Cys115 | C*YDPDFEKLKPEY^3+^ | y_6_: *m/z* 599.3→777.5 (30), y_10_^2+^: *m/z* 599.3→633.3 (27), y_12_^2+^: *m/z* 599.3→690.8 (25) and a_2_: *m/z* 599.3→388.1 (30) |
|  | Cys174 | ERNQVFEPSC*L^2+^ | b_6_: *m/z* 735.8→774.4 (30), b_7_: *m/z* 735.8→903.4 (30), b_9_: *m/z* 735.8→1087.5 (30) and y_4_: *m/z* 735.8→568.2 (30) |
|  |  | RNQVFEPSC*L^2+^ | b_6_: *m/z* 671.3→774.4 (27), b_8_: *m/z* 671.3→958.5 (30), b_9_: *m/z* 671.3→1210.5 (30) and y_4_: *m/z* 671.3→568.2 (25) |
|  |  | DVLERNQVFEPSC*L^2+^ | b_8_: *m/z* 842.9→954.5 (30), b_9_: *m/z* 842.9→1101.6 (30), b_10_: *m/z* 842.9→1230.6 (30) and y_3_: *m/z* 842.9→455.2 (30) |
| P1 | Cys15 | PVRGRC*AALRM^3+^ | y_4_: *m/z* 460.2→490.3 (32), y_5_: *m/z* 460.2→561.3 (30), (y_10_−NH_3_)^2+^: *m/z* 460.2→632.8 (27) and (b_10_+H_2_O)^3+^: *m/z* 460.2→416.6 (27) |
|  |  | VYFPVRGRC*AAL^3+^ | y_9_^2+^: *m/z* 500.9→546.3 (22), y_10_^2+^: *m/z* 500.9→619.8 (22), y_11_^2+^: *m/z* 500.9→701.4 (23) and a_2_: *m/z* 500.9→235.1 (23) |
|  |  | VYFPVRGRC*^3+^ | y_5_−NH_3_: *m/z* 415.9→722.3 (32), y_7_^2+^: *m/z* 415.9→722.3 (18), y_8_^2+^: *m/z* 415.9→722.3 (28) and a_2_: *m/z* 415.9→722.3 (28) |
|  | Cys48 | VETWQEGSLKASC*L^2+^ | y_8_: *m/z* 850.4→927.5 (32), y_9_: *m/z* 850.4→1056.5 (32), y_10_: *m/z* 850.4→1184.6 (32) y_2_: *m/z* 850.4→384.2 (32) |
|  |  | TWQEGSLKASC*L^2+^ | y_8_: *m/z* 736.3→927.5 (32), y_9_: *m/z* 736.3→1056.5 (28), y_10_: *m/z* 736.3→1184.6 (28) and a_2_: *m/z* 736.3→260.1 (27) |

**Table S2** Continued.

| **GST** | **Site** | **Peptide precursor ion** | **Fragment ion: transition (CE [V])** |
| --- | --- | --- | --- |
|  |  | **Pepsin digestion** |  |
| P1 | Cys48 | TVETWQEGSLKASC*L^3+^ | y_7_: *m/z* 601.0→870.4 (28), y_8_: *m/z* 601.0→927.5 (23), y_9_: *m/z* 601.0→1056.5 (27) and y_2_: *m/z* 601.0→384.2 (23) |
|  | Cys102 | GVEDLRC*KYISL^3+^ | y_8_^2+^: *m/z* 515.6→572.8 (27), y_9_^2+^: *m/z* 515.6→630.3 (28), y_10_^2+^: *m/z* 515.6→694.8 (28) and y_2_: *m/z* 515.6→219.1 (32) |
|  |  | GVEDLRC*KY^2+^ | y_5_: *m/z* 616.3→831.4 (32), y_6_: *m/z* 616.3→946.4 (32), y_7_: *m/z* 616.3→1075.5 (32) and y_7_^2+^: *m/z* 616.3→538.2 (28) |
|  |  | VDMVNDGVEDLRC*KYISL^3+^ | y_14_^2+^: *m/z* 740.0→887.4 (30), y_15_^2+^: *m/z* 740.0→937.0 (32), y_16_^2+^: *m/z* 740.0→1002.5 (28) and b_3_: *m/z* 740.0→346.1 (32) |
|  | Cys170 | IHEVLAPGC*L^2+^ | y_5_: *m/z* 600.8→609.3 (23), b_6_: *m/z* 600.8→663.4 (23), b_8_: *m/z* 600.8→817.5 (27) and y_4_: *m/z* 600.8→538.2 (23) |
|  |  | LLIHEVLAPGC*L^2+^ | b_7_: *m/z* 713.9→818.5 (27), b_8_: *m/z* 713.9→889.6 (23), b_10_: *m/z* 713.9→1043.6 (28) and y_4_: *m/z* 713.9→538.2 (27) |
|  |  | LIHEVLAPGC*LDA^2+^ | y_7_: *m/z* 750.4→795.3 (28), b_9_: *m/z* 750.4→930.5 (32), b_10_: *m/z* 750.4→1182.6 (30) and y_4_: *m/z* 750.4→724.3 (28) |

**Table S3** List of identified (peptide confidence >95%) carbamidomethylated (CAM) cysteines in GSTA1, M1, M2 and P1.

| **GST** | **Cys** | **Peptide** | **Protease** | **z** |
| --- | --- | --- | --- | --- |
| A1 | 112 | ALIDMYIEGIADLGEMILLLPVC(CAM)PPEEK | Pepsin and trypsin | +3 and +4 |
|  |  | IEGIADLGEMILLLPVC(CAM)PPEEK | Trypsin | +3 |
|  |  | ILLLPVC(CAM)PPEEK | Trypsin | +2 and +3 |
|  |  | LPVC(CAM)PPEEK | Trypsin | +2 |
|  |  | LPVC(CAM)PPEEKD | Pepsin | +2 |
|  |  | LPVC(CAM)PPEEKDAKL | Pepsin | +2 and +3 |
|  |  | LPVC(CAM)PPEEKDAKLAL | Pepsin | +3 |
|  |  | LLPVC(CAM)PPEEK | Trypsin | +2 |
|  |  | LLPVC(CAM)PPEEKD | Pepsin | +2 |
|  |  | LLPVC(CAM)PPEEKDAKL | Pepsin | +2 and +3 |
|  |  | LLPVC(CAM)PPEEKDAKLAL | Pepsin | +3 |
|  |  | PVC(CAM)PPEEK | Trypsin | +1 and +2 |
|  |  | PVC(CAM)PPEEKD | Pepsin | +2 |
|  |  | PVC(CAM)PPEEKDAKL | Pepsin | +2 and +3 |
|  |  | PVC(CAM)PPEEKDAKLAL | Pepsin | +2 and +3 |
|  |  | YIEGIADLGEMILLLPVC(CAM)PPEEK | Trypsin | +3 and +4 |
| M1 | Cys78 | ITQSNAILC(CAM)Y | Trypsin | +1 and +2 |
|  |  | ITQSNAILC(CAM)YIAR | Trypsin | +2 and +3 |
|  |  | TQSNAILC(CAM)Y | Trypsin | +1 |
|  | Cys78 and 87 | C(CAM)YIARKHNLC(CAM)GETEE | Pepsin | +3 |
|  | Cys87 | HNLC(CAM)GETEEEKIR | Trypsin | +3 |

**Table S3** Continued.

| **GST** | **Cys** | **Peptide** | **Protease** | **z** |
| --- | --- | --- | --- | --- |
| M1 | Cys87 | KHNLC(CAM)GETEEEKIR | Trypsin | +4 |
|  |  | YIARKHNLC(CAM)GETEEE | Pepsin | +3 and +4 |
|  | Cys115 | DNHMQLGMIC(CAM)YNPEFEK | Trypsin | +3 |
|  |  | GMIC(CAM)YNPEFEK | Trypsin | +2 |
|  |  | IC(CAM)YNPEFEK | Trypsin | +2 |
|  |  | IRVDILENQTMDNHMQLGMIC(CAM)YNPEFEK | Trypsin | +3, +4 and +5 |
|  |  | LGMIC(CAM)YNPEFEK | Trypsin | +2 |
|  |  | MQLGMIC(CAM)YNPEFEK | Trypsin | +2 |
|  |  | NHMQLGMIC(CAM)YNPEFEK | Trypsin | +3 |
|  |  | QLGMIC(CAM)YNPEFEK | Trypsin | +2 |
|  |  | QTMDNHMQLGMIC(CAM)YNPEFEK | Trypsin | +3 |
|  |  | VDILENQTMDNHMQLGMIC(CAM)Y | Trypsin | +3 |
|  |  | VDILENQTMDNHMQLGMIC(CAM)YNPEFEK | Trypsin | +3 and +4 |
|  | Cys174 | C(CAM)LDAFPNLK | Trypsin | +1 and +2 |
|  |  | C(CAM)LDAFPNLKDFISR | Trypsin | +2 and +3 |
| M2 | Cys87 | HNLC(CAM)GESEKEQIR | Trypsin | +3 and +4 |
|  |  | HNLC(CAM)GESEKEQIREDILENQFMDSR | Trypsin | +3 and +4 |
|  |  | IARKHNLC(CAM)GESEKEQIRE | Pepsin | +4 |
|  |  | KHNLC(CAM)GESEKEQIREDILE | Trypsin | +4 |
|  |  | KHNLC(CAM)GESEKEQIREDILEN | Trypsin | +4 |
|  |  | KHNLC(CAM)GESEKEQIREDILENQFMDSR | Trypsin | +3, +4, +5 and +6 |

**Table S3** Continued.

| **GST** | **Cys** | **Peptide** | **Protease** | **z** |
| --- | --- | --- | --- | --- |
| M2 | Cys87 | LC(CAM)GESEKEQIREDILENQFMDSR | Trypsin | +4 |
|  |  | NLC(CAM)GESEKEQIREDILENQFMDSR | Trypsin | +4 |
|  |  | YIARKHNLC(CAM)GESEKEQIRE | Pepsin | +5 |
|  | Cys115 | AKLC(CAM)YDPDF | Pepsin | +1 and +2 |
|  |  | AKLC(CAM)YDPDFE | Pepsin | +2 |
|  |  | AKLC(CAM)YDPDFEKLKPEY | Pepsin | +4 |
|  |  | AKLC(CAM)YDPDFEKLKPEYL | Pepsin | +3 |
|  |  | AKLC(CAM)YDPDFEKLKPEYLQA | Pepsin | +4 |
|  |  | C(CAM)YDPDFEK | Trypsin | +1 and +2 |
|  |  | C(CAM)YDPDFEKLKPEY | Pepsin | +2 and +3 |
|  |  | C(CAM)YDPDFEKLKPEYL | Pepsin | +2 and +3 |
|  |  | C(CAM)YDPDFEKLKPEYLQA | Pepsin | +3 |
|  |  | C(CAM)YDPDFEKLKPEYLQAL | Pepsin | +3 |
|  |  | C(CAM)YDPDFEKLKPEYLQALPEM | Pepsin | +3 |
|  |  | KLC(CAM)YDPDFEK | Trypsin | +3 |
|  |  | LAKLC(CAM)YDPDF | Pepsin | +2 |
|  |  | LC(CAM)YDPDF | Trypsin | +2 |
|  |  | LC(CAM)YDPDFEK | Trypsin | +1, +2 and +3 |
|  |  | LC(CAM)YDPDFEKLKPEYL | Trypsin | +3 |
|  |  | LC(CAM)YDPDFEKLKPEYLQALPEML | Trypsin | +3 |
|  |  | LC(CAM)YDPDFEKLKPEYLQALPEMLK | Trypsin | +3 and +4 |

**Table S3** Continued.

| **GST** | **Cys** | **Peptide** | **Protease** | **z** |
| --- | --- | --- | --- | --- |
| M2 | Cys174 | C(CAM)LDAFPNLK | Trypsin | +1 and +2 |
|  |  | C(CAM)LDAFPNLKDFISR | Trypsin | +2 and +3 |
|  |  | DVLERNQVFEPSC(CAM) | Pepsin | +2 and +3 |
|  |  | DVLERNQVFEPSC(CAM)L | Pepsin | +2 and +3 |
|  |  | EPSC(CAM)LDAFPNLK | Trypsin | +2 |
|  |  | ERNQVFEPSC(CAM) | Pepsin | +2 |
|  |  | ERNQVFEPSC(CAM)L | Pepsin | +2 and +3 |
|  |  | LERNQVFEPSC(CAM)L | Pepsin | +2 |
|  |  | NQVFEPSC(CAM)L | Trypsin | +1 and +2 |
|  |  | NQVFEPSC(CAM)LD | Trypsin | +2 |
|  |  | NQVFEPSC(CAM)LDAFPN | Trypsin | +2 and +3 |
|  |  | NQVFEPSC(CAM)LDAFPNLK | Trypsin | +2, +3 and +4 |
|  |  | NQVFEPSC(CAM)LDAFPNLKDF | Trypsin | +2 and +3 |
|  |  | NQVFEPSC(CAM)LDAFPNLKDFISR | Trypsin | +2, +3 and +4 |
|  |  | NQVFEPSC(CAM)LDAFPNLKDFISRFEGLEK | Trypsin | +3 and +4 |
|  |  | PSC(CAM)LDAFPN | Trypsin | +1 |
|  |  | PSC(CAM)LDAFPNLK | Trypsin | +2 |
|  |  | PSC(CAM)LDAFPNLKDFISR | Trypsin | +2 |
|  |  | QVFEPSC(CAM)LDAFPNLK | Trypsin | +2 |
|  |  | VFEPSC(CAM)LDAFPNLK | Trypsin | +2 and +3 |
|  |  | VFEPSC(CAM)LDAFPNLKDFISR | Trypsin | +3 |

**Table S3** Continued.

| **GST** | **Cys** | **Peptide** | **Protease** | **z** |
| --- | --- | --- | --- | --- |
| M2 | Cys174 | VLERNQVFEPSC(CAM) | Pepsin | +2 |
|  |  | VLERNQVFEPSC(CAM)L | Pepsin | +2 |
| P1 | Cys48 | ASC(CAM)LYGQLPK | Pepsin and trypsin | +1, +2 and +3 |
|  |  | C(CAM)LYGQLPK | Trypsin | +2 |
|  |  | QEGSLKASC(CAM)L | Pepsin | +2 |
|  |  | SC(CAM)LYGQLPK | Trypsin | +1 and +2 |
|  |  | TWQEGSLKASC(CAM)L | Pepsin | +2 |
|  |  | VETWQEGSLKASC(CAM)L | Pepsin | +2 |
|  |  | WQEGSLKASC(CAM)L | Pepsin | +2 |
|  | Cys170 | ADYNLLDLLLIHEVLAPGC(CAM)LDAFPLL | Trypsin | +3 |
|  |  | ADYNLLDLLLIHEVLAPGC(CAM)LDAFPLLS | Trypsin | +3 |
|  |  | ADYNLLDLLLIHEVLAPGC(CAM)LDAFPLLSAY | Trypsin | +3 |
|  |  | APGC(CAM)LDAFPLL | Trypsin | +2 |
|  |  | APGC(CAM)LDAFPLLS | Trypsin | +2 |
|  |  | C(CAM)LDAFPLL | Trypsin | +1 |
|  |  | DLLLIHEVLAPGC(CAM)LD | Trypsin | +2 and +3 |
|  |  | DLLLIHEVLAPGC(CAM)LDAFPLL | Trypsin | +3 |
|  |  | DLLLIHEVLAPGC(CAM)LDAFPLLS | Trypsin | +2 and +3 |
|  |  | DLLLIHEVLAPGC(CAM)LDAFPLLSAY | Trypsin | +3 |
|  |  | EVLAPGC(CAM)LDAFPLL | Trypsin | +2 |
|  |  | GC(CAM)LDAFPLLS | Trypsin | +2 |

**Table S3** Continued.

| **GST** | **Cys** | **Peptide** | **Protease** | **z** |
| --- | --- | --- | --- | --- |
| P1 | Cys170 | GC(CAM)LDAFPLLSAY | Trypsin | +2 |
|  |  | HEVLAPGC(CAM)LDAFPLL | Trypsin | +2 |
|  |  | IHEVLAPGC(CAM)L | Pepsin and trypsin | +2 |
|  |  | IHEVLAPGC(CAM)LD | Trypsin | +2 |
|  |  | IHEVLAPGC(CAM)LDA | Pepsin | +2 |
|  |  | IHEVLAPGC(CAM)LDAFPL | Trypsin | +2 |
|  |  | IHEVLAPGC(CAM)LDAFPLL | Trypsin | +2 and +3 |
|  |  | IHEVLAPGC(CAM)LDAFPLLS | Trypsin | +2 and +3 |
|  |  | IHEVLAPGC(CAM)LDAFPLLSAY | Trypsin | +2 and +3 |
|  |  | LDLLLIHEVLAPGC(CAM)LDAFPLL | Trypsin | +2 and +3 |
|  |  | LDLLLIHEVLAPGC(CAM)LDAFPLLS | Trypsin | +2 and +3 |
|  |  | LDLLLIHEVLAPGC(CAM)LDAFPLLSAY | Trypsin | +3 |
|  |  | LIHEVLAPGC(CAM)L | Pepsin | +2 |
|  |  | LIHEVLAPGC(CAM)LD | Trypsin | +2 |
|  |  | LIHEVLAPGC(CAM)LDA | Pepsin | +2 |
|  |  | LIHEVLAPGC(CAM)LDAFPL | Trypsin | +2 |
|  |  | LIHEVLAPGC(CAM)LDAFPLL | Trypsin | +2 and +3 |
|  |  | LIHEVLAPGC(CAM)LDAFPLLS | Trypsin | +2 and +3 |
|  |  | LIHEVLAPGC(CAM)LDAFPLLSAY | Trypsin | +2 and +3 |
|  |  | LLDLLLIHEVLAPGC(CAM)LD | Trypsin | +2 and +3 |
|  |  | LLDLLLIHEVLAPGC(CAM)LDAFP | Trypsin | +2 and +3 |

**Table S3** Continued.

| **GST** | **Cys** | **Peptide** | **Protease** | **z** |
| --- | --- | --- | --- | --- |
| P1 | Cys170 | LLDLLLIHEVLAPGC(CAM)LDAFPL | Trypsin | +3 |
|  |  | LLDLLLIHEVLAPGC(CAM)LDAFPLL | Trypsin | +2 and +3 |
|  |  | LLDLLLIHEVLAPGC(CAM)LDAFPLLS | Trypsin | +3 and +4 |
|  |  | LLDLLLIHEVLAPGC(CAM)LDAFPLLSAY | Trypsin | +3 and +4 |
|  |  | LLIHEVLAPGC(CAM)LD | Trypsin | +2 |
|  |  | LLIHEVLAPGC(CAM)LDAFPLLS | Trypsin | +2 |
|  |  | NLLDLLLIHEVLAPGC(CAM)L | Trypsin | +3 |
|  |  | NLLDLLLIHEVLAPGC(CAM)LDAFPLL | Trypsin | +3 |
|  |  | TFIVGDQISFADYNLLDLLLIHEVLAPGC(CAM)LDAFPLL | Trypsin | +4 |
|  |  | TFIVGDQISFADYNLLDLLLIHEVLAPGC(CAM)LDAFPLLS | Trypsin | +4 |
|  |  | TFIVGDQISFADYNLLDLLLIHEVLAPGC(CAM)LDAFPLLSAY | Trypsin | +4 |
|  |  | VLAPGC(CAM) | Pepsin | +1 |
|  |  | VLAPGC(CAM)LDAFPL | Trypsin | +2 |
|  |  | VLAPGC(CAM)LDAFPLL | Trypsin | +2 |
|  |  | VLAPGC(CAM)LDAFPLLS | Trypsin | +2 |
|  |  | VLAPGC(CAM)LDAFPLLSAY | Trypsin | +2 and +3 |


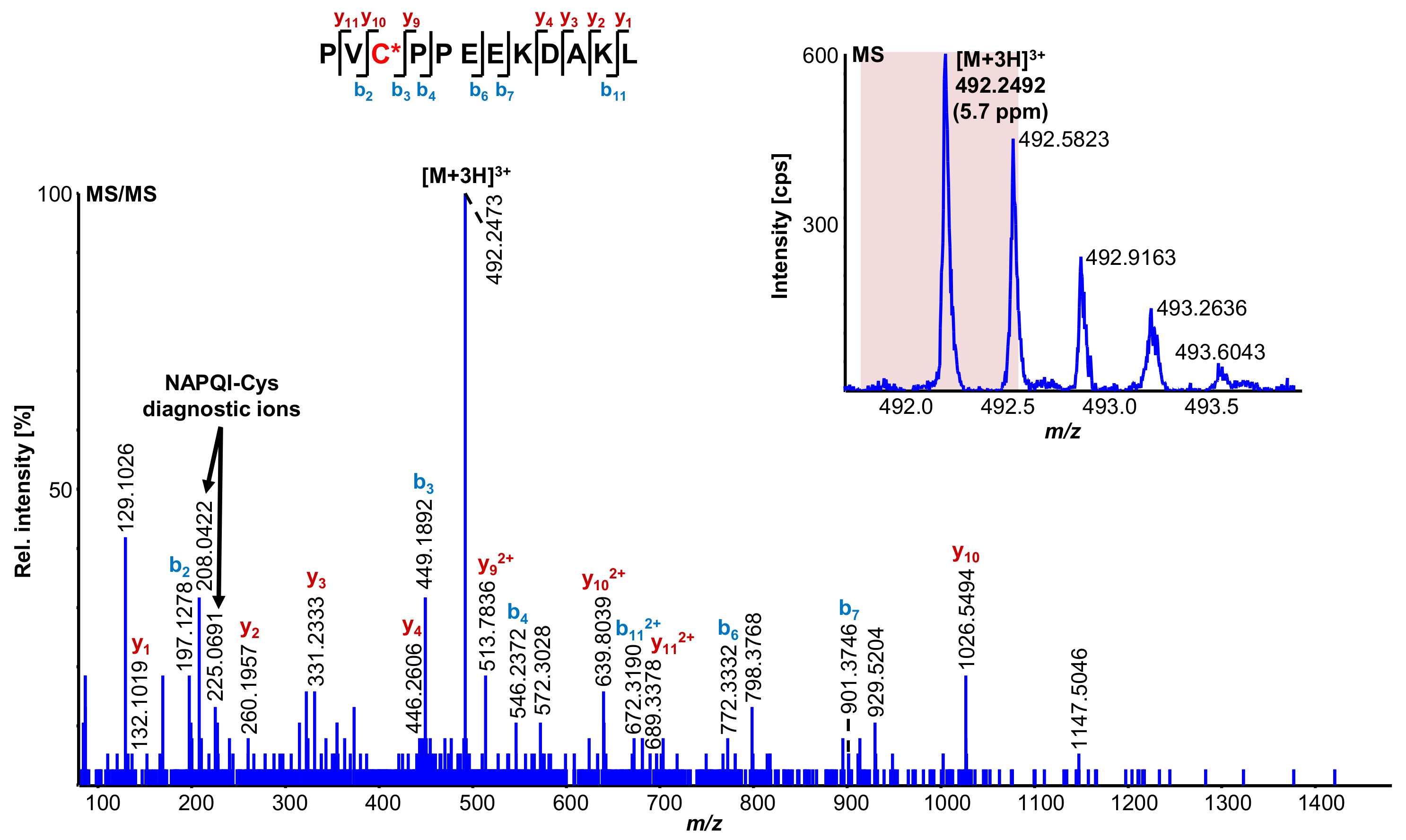


**Figure S1A** DDA spectra of NAPQI-GSTA1 peptic peptide PVC*PPEEKDAKL (acquired at 14.8 min).


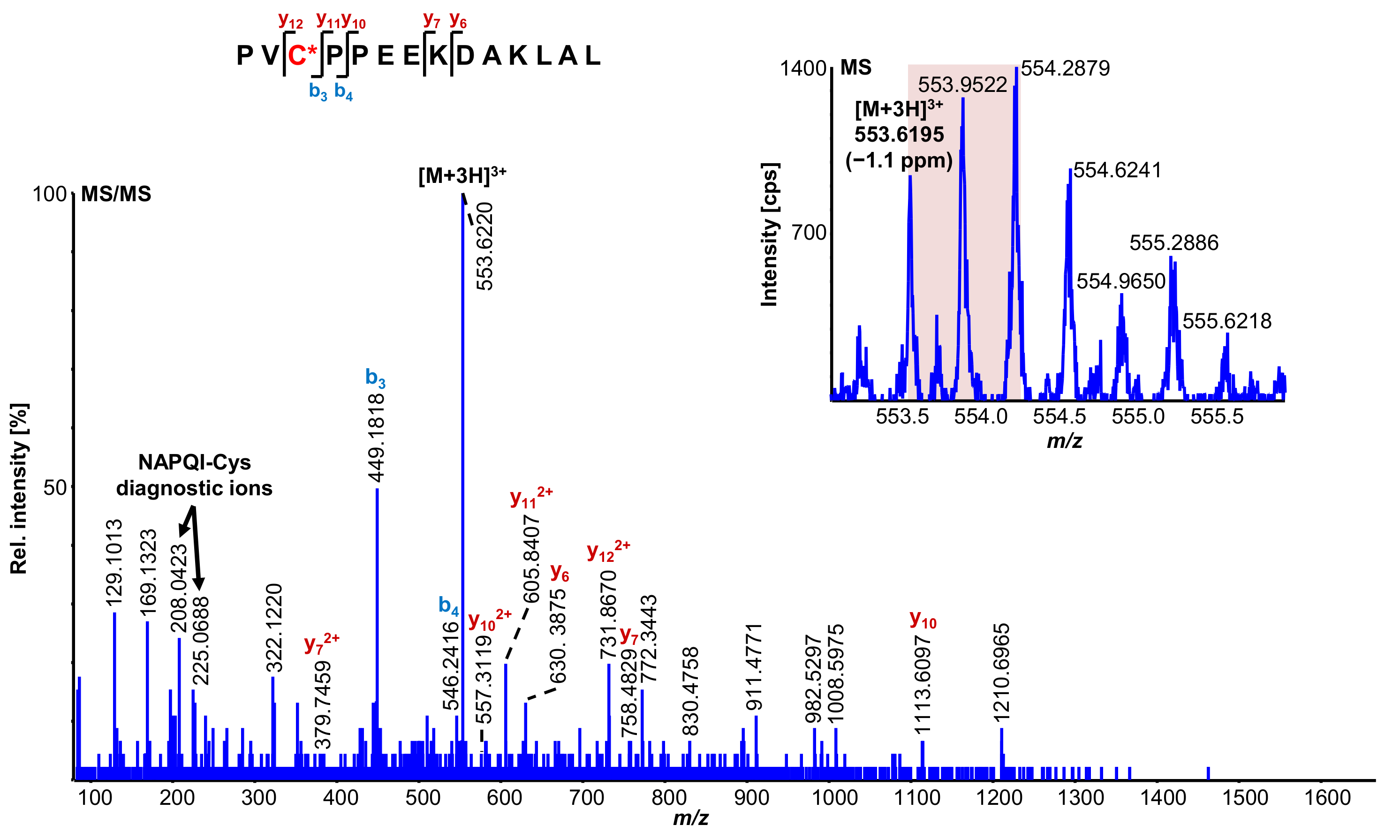


**Figure S1B** DDA spectra of NAPQI-GSTA1 peptic peptide PVC*PPEEKDAKLAL (acquired at 19.6 min).


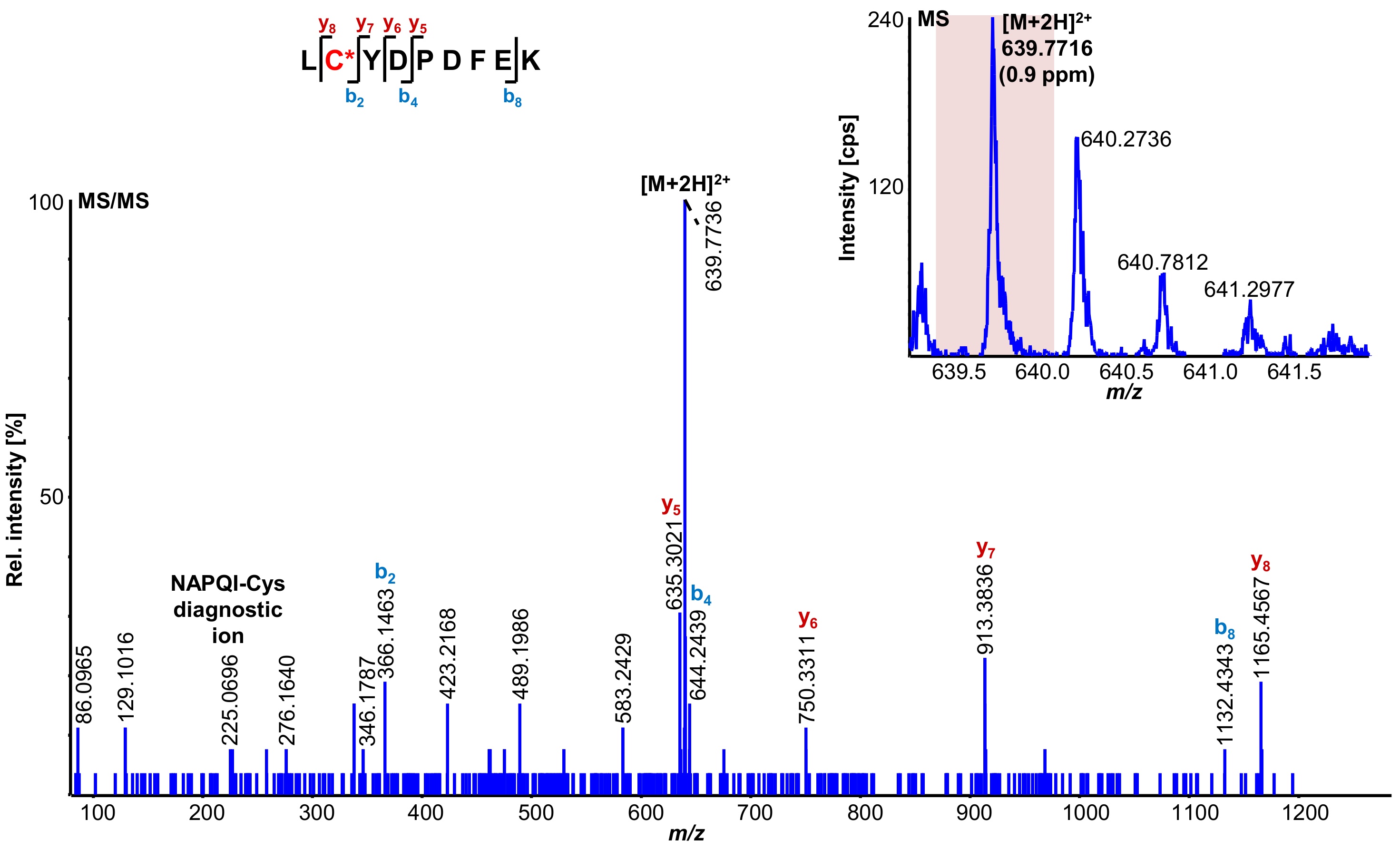


**Figure S1C** DDA spectra of NAPQI-GSTM2 tryptic peptide LC*YDPDFEK (acquired at 20.3 min).


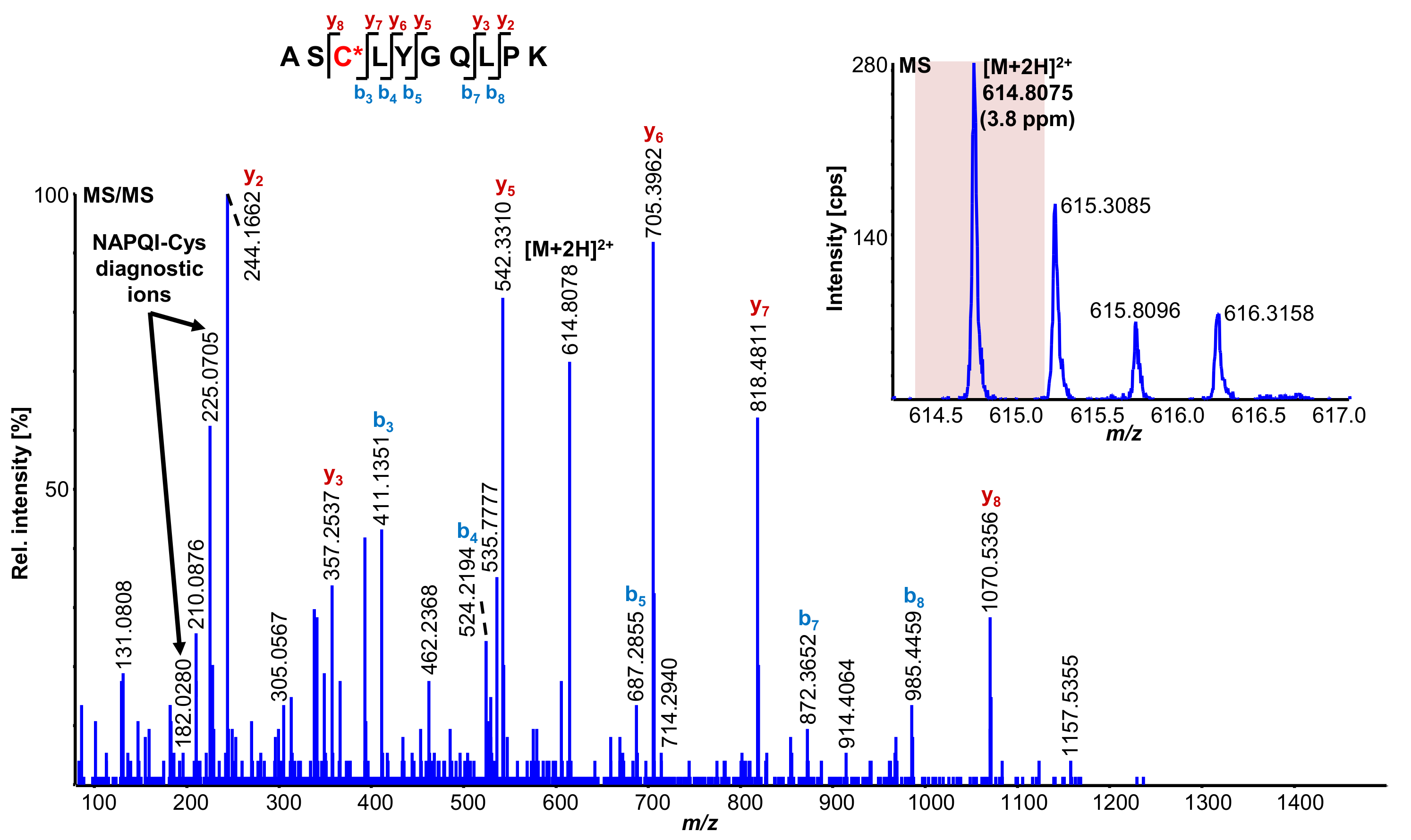


**Figure S1D** DDA spectra of NAPQI-GSTP1 tryptic peptide ASC*LYGQLPK (acquired at 19.7 min).


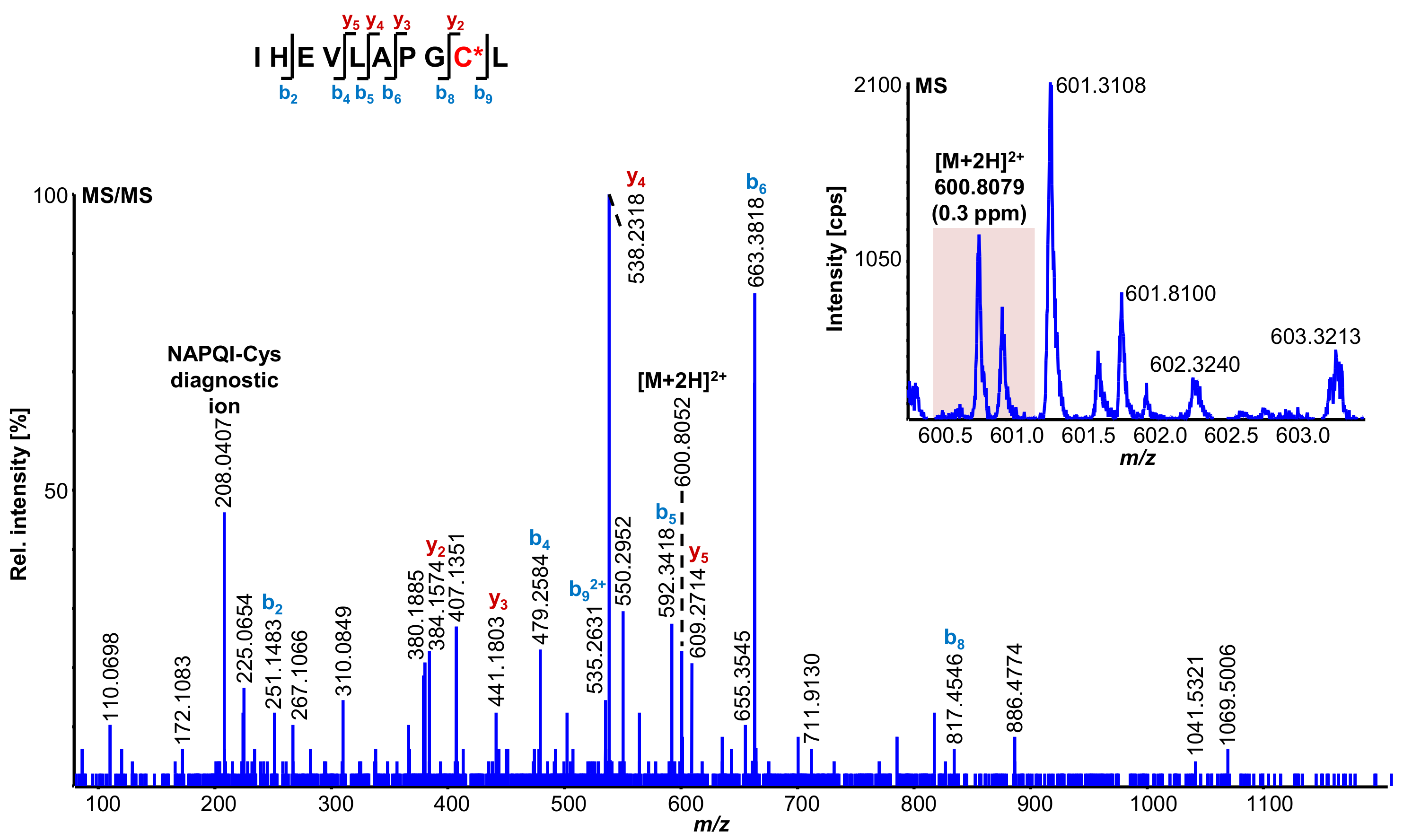


**Figure S1E** DDA spectra of NAPQI-GSTP1 peptic peptide IHEVLAPGC*L (acquired at 23.5 min).


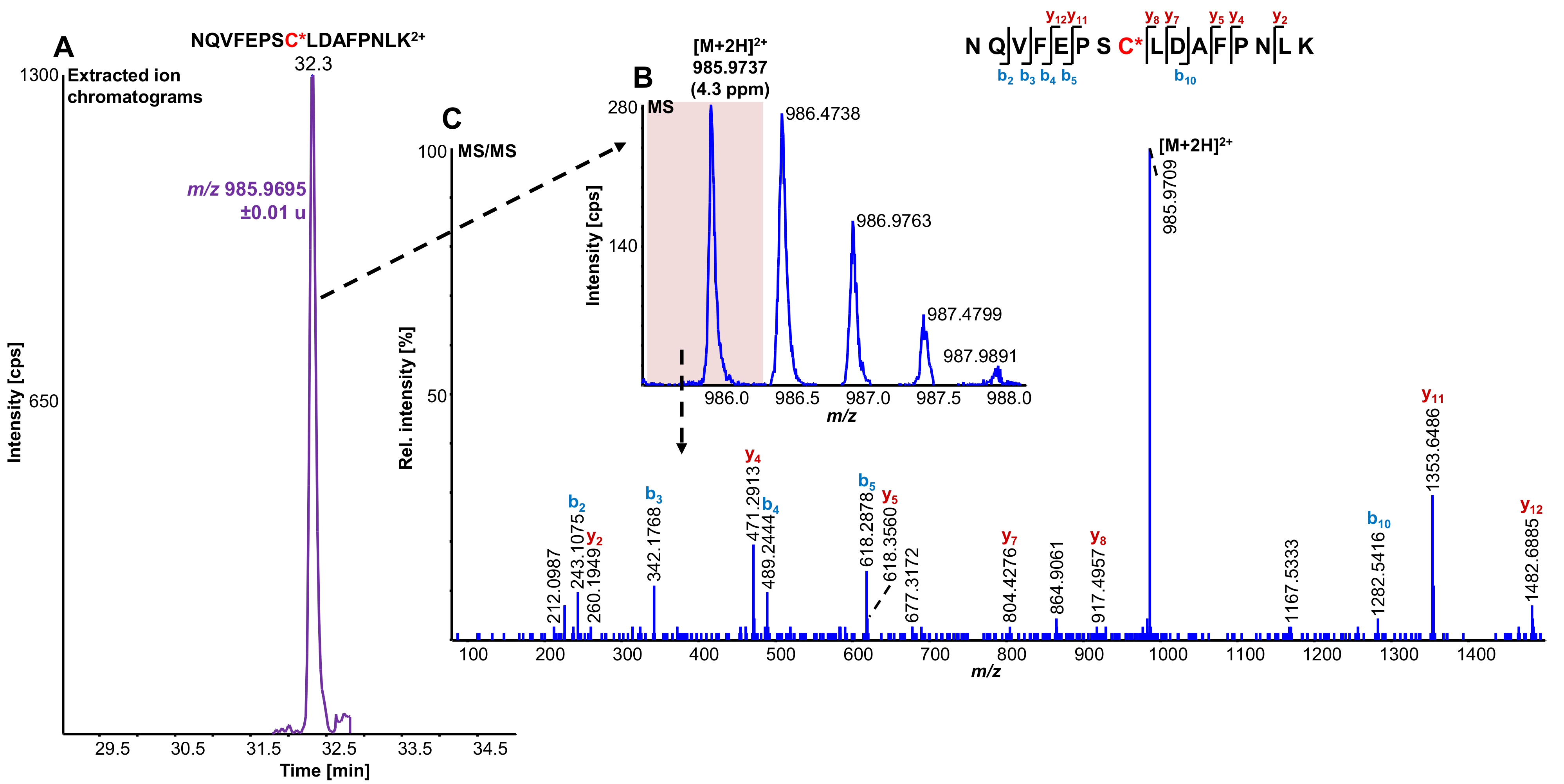


**Figure S2** Extracted ion chromatogram of NAPQI-GSTM2 tryptic peptide NQVFEPSC*LDAFPNLK **(A)**, including survey scan MS **(B)** and triggered MS/MS **(C)**.


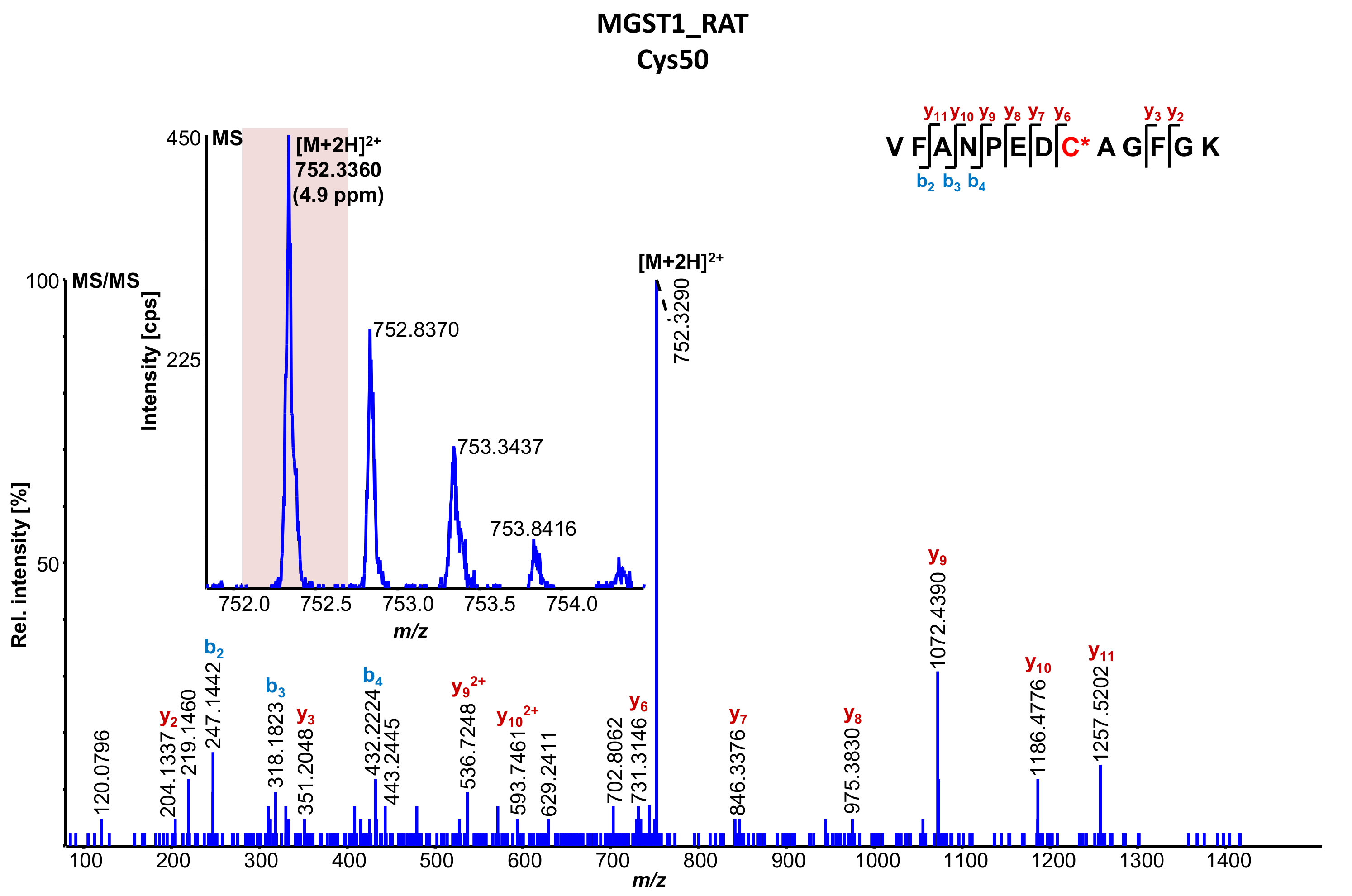


**Figure S3** DDA spectra of NAPQI-rat MGST1 tryptic peptide VFANPEDC*AGFGK (acquired at 22.0 min).


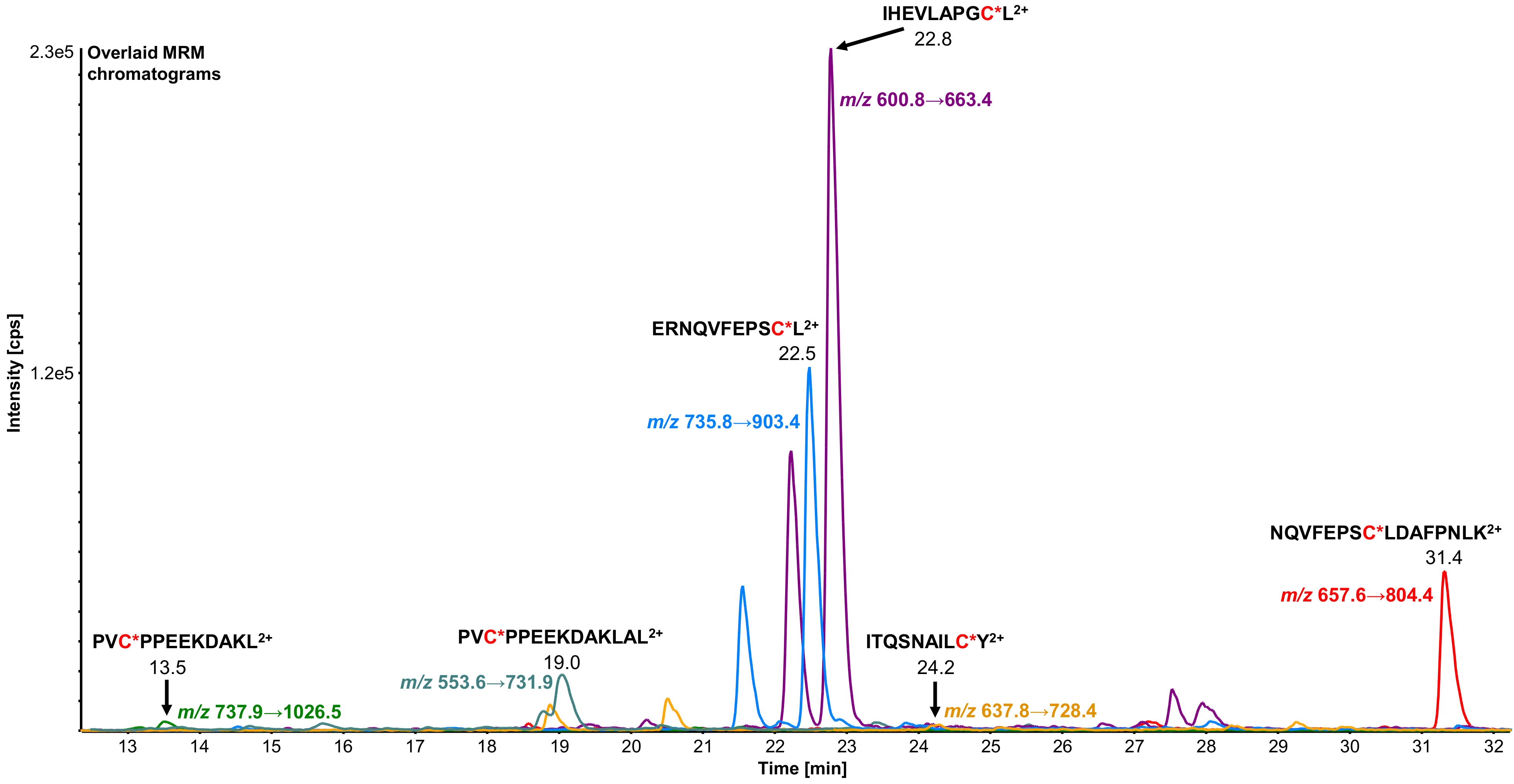


**Figure S4** Overlaid MRM chromatograms (60 min LC) of found modified peptides using RLM incubation and SPE purification.


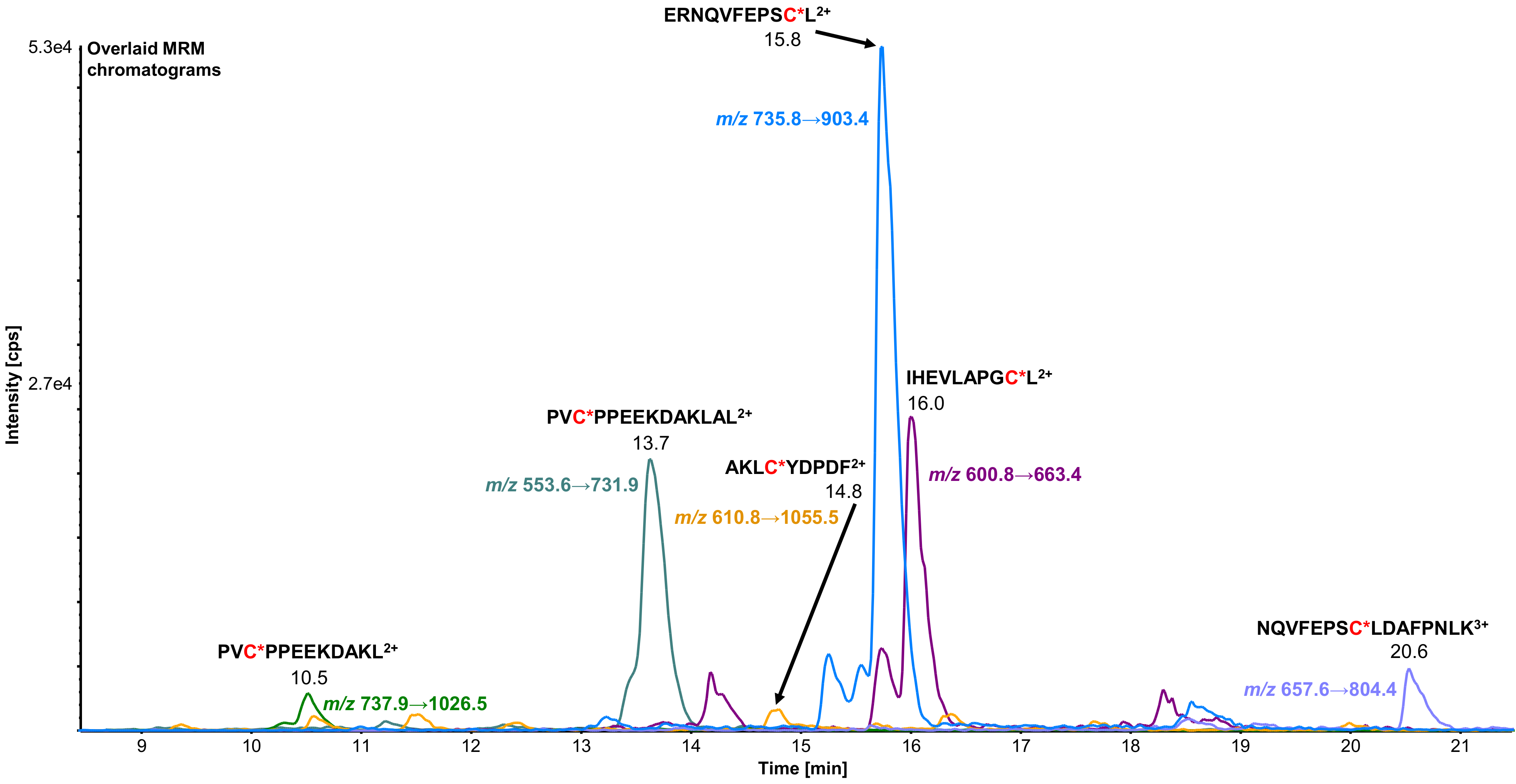


**Figure S5** Overlaid MRM chromatograms (30 min LC) of found modified peptides using RLM incubation, SPE purification and high-pH RP fractionation.


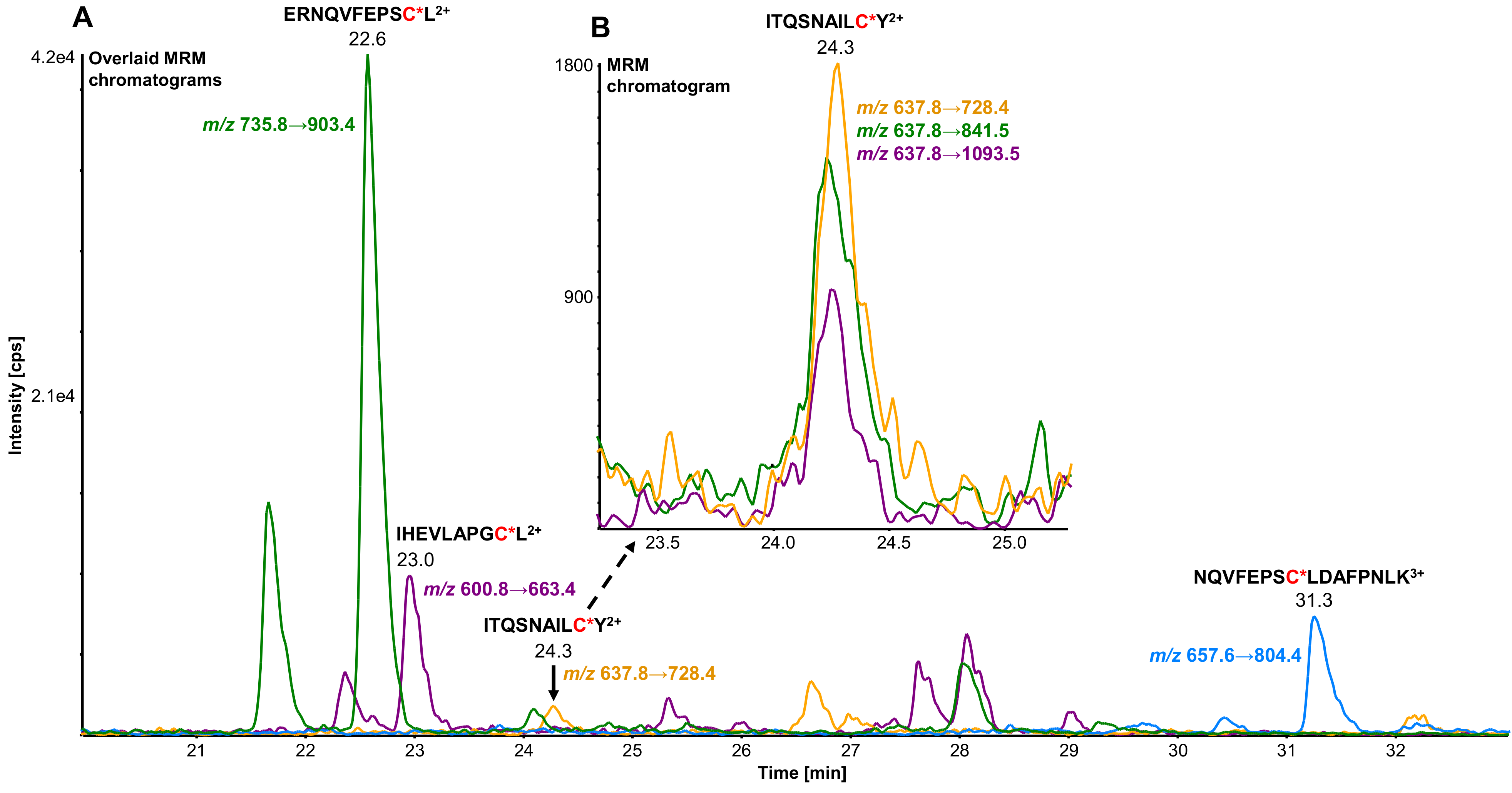


**Figure S6** Overlaid MRM chromatograms (60 min LC) of found modified peptides using CYP3A4 Supersomes incubation and SPE purification **(A)**, including peptide ITQSNAILC*Y **(B)**.


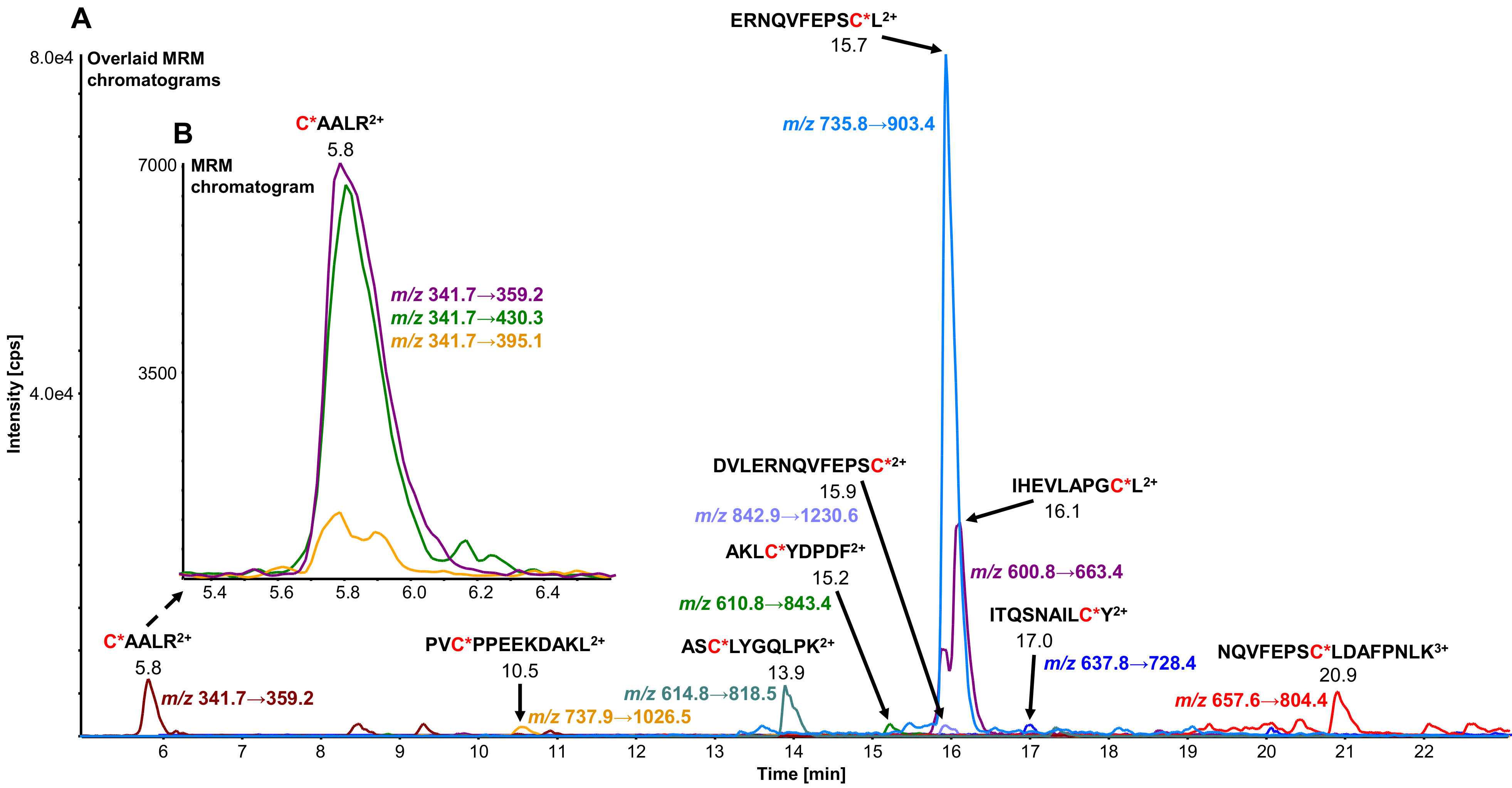


**Figure S7** Overlaid MRM chromatograms (30 min LC) of found modified peptides using CYP3A4 Supersomes incubation, SPE purification and high-pH RP fractionation **(A)**, including peptide C*AALR **(B)**.


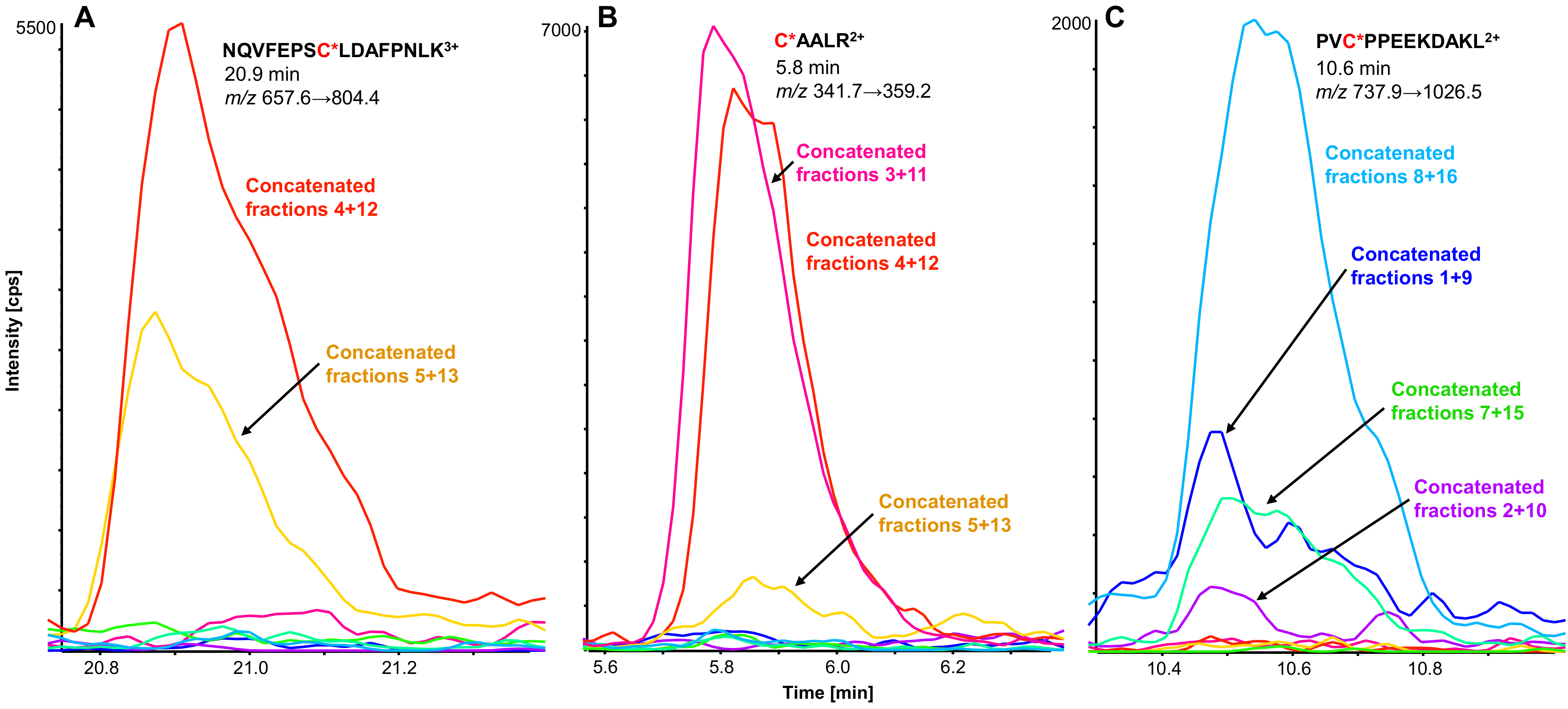


**Figure S8** Distribution of selected MRM transitions over all concatenated fractions of peptides NQVFEPSC*LDAFPNLK **(A)**, C*AALR **(B)** and PVC*PPEEKDAKL **(C)**, showing individual differences in elution during high-pH RP fractionation.
